# Pilus Phase Variation Switches Gonococcal Adherence to Invasion by Caveolin-1-Dependent Host Cell Signaling

**DOI:** 10.1371/journal.ppat.1003373

**Published:** 2013-05-23

**Authors:** Michaela Faulstich, Jan-Peter Böttcher, Thomas F. Meyer, Martin Fraunholz, Thomas Rudel

**Affiliations:** 1 Chair of Microbiology, University of Würzburg Biocenter, Würzburg, Germany; 2 Max Planck Institute for Infection Biology, Dept. Molecular Biology, Berlin, Germany; Faculté de Médecine Paris Descartes Necker, France

## Abstract

Many pathogenic bacteria cause local infections but occasionally invade into the blood stream, often with fatal outcome. Very little is known about the mechanism underlying the switch from local to invasive infection. In the case of *Neisseria gonorrhoeae*, phase variable type 4 pili (T4P) stabilize local infection by mediating microcolony formation and inducing anti-invasive signals. Outer membrane porin PorB_IA_, in contrast, is associated with disseminated infection and facilitates the efficient invasion of gonococci into host cells. Here we demonstrate that loss of pili by natural pilus phase variation is a prerequisite for the transition from local to invasive infection. Unexpectedly, both T4P-mediated inhibition of invasion and PorB_IA_-triggered invasion utilize membrane rafts and signaling pathways that depend on caveolin-1-Y14 phosphorylation (Cav1-pY14). We identified p85 regulatory subunit of PI3 kinase (PI3K) and phospholipase Cγ1 as new, exclusive and essential interaction partners for Cav1-pY14 in the course of PorB_IA_-induced invasion. Active PI3K induces the uptake of gonococci via a new invasion pathway involving protein kinase D1. Our data describe a novel route of bacterial entry into epithelial cells and offer the first mechanistic insight into the switch from local to invasive gonococcal infection.

## Author Summary

*Neisseria gonorrhoeae* is a human-specific bacterial pathogen causing gonorrhea. With over 100 million infections per year it is among the most prevalent sexually-transmitted diseases worldwide. Whereas most infections are localized, occasionally *N. gonorrhoeae* invades the blood stream. The resulting disseminated infections often lead to serious conditions such as dermatitis, sepsis, endocarditis, and arthritis. Gonorrhea causes particular concern due to the currently ongoing dramatic spread of multi-resistant bacteria, which might render the disease untreatable in the future. Here, we describe molecular events that lead to the switch from local to invasive gonococcal infections. Whereas pili constitute adhesive structures leading to localized infections, the natural loss of piliation unblocks a hitherto unidentified signaling cascade initiated by the interaction of an outer membrane porin and a eukaryotic scavenger receptor. We show that in both cases the different infection outcomes rely on distinct signaling molecules, which are either recruited to or displaced from caveolae. Furthermore, we unravel the signaling network which activates cytoskeletal rearrangements that ultimately lead to the porin/scavenger receptor-triggered invasion of the host cell.

## Introduction

The human-specific Gram-negative bacterium *Neisseria gonorrhoeae* is the cause of the sexually-transmitted disease gonorrhea. With more than 106 million infections per year (source: WHO) it presents a serious threat to world health. Moreover, the current global dramatic spread of multi-resistant gonococci and the predicted impact of untreatable gonorrhea on HIV transmission [1] has alarmed the WHO and initiated the release of a global action plan in 2012 to control the spread and impact of multi-resistant gonococci (http://www.who.int/en). Besides causing local infections, gonococci may also spread within the host. These systemic disseminated gonococcal infections (DGI) lead to serious conditions such as dermatitis, sepsis, endocarditis, and arthritis [2], [3].

*N. gonorrhoeae* strains possess the ability to form type IV pili (T4P), which establish an initial contact and adherence to the host cell. Subsequently, gonococci utilize Opacity-associated (opa) proteins to intimately bind to and invade into host cells [4], [5], [6], [7]. Within the 11 members of the Opa protein family, the Opa_50_ protein binds to heparan sulfate proteoglycans (HSPG) [8], [9] or fibronectin and integrins [10] whereas all other Opa proteins (Opa_51-60_) target members of the carcinoembryonic antigen-related cellular adhesion molecules (CEACAM; for review see [11]). Another route to enter primary cervical epithelial cells requires the cooperative binding of the major outer membrane protein PorB, pili and lipooligosaccharide to the complement receptor type 3 [12], [13]. Finally, entry of *N. gonorrhoeae* into non-professional phagocytes is mediated by PorB subtype A (PorB_IA_). By contrast, the closely related subtype B (PorB_IB_) does not confer internalization [10], [14]. This invasion mechanism is phosphate-sensitive and independent of pili and Opa-proteins. We recently resolved the structure of PorB_IA_ and identified Arg/His92 as critical for phosphate binding and invasion [15]. Exchange of Arg/His92 highly conserved in PorB_IA_ from DGI strains for Ser, the respective amino acid found invariantly in all PorB_IB_ subtypes, leads to the loss of the invasive phenotype via this otherwise fully functional porin [15]. This high degree of structural conservation of invasive versus non-invasive PorB from strains associated with disseminated versus local infection, respectively, support a role of PorB_IA_ in DGI. Low-phosphate conditions are found in the blood stream and may thus allow the unmasking of a receptor-interacting region in PorB_IA_ [15]. While strains expressing PorB_IB_ can occasionally disseminate, PorB_IA_-expressing gonococci are clearly overrepresented in systemic infections (20% in all versus 80% in DGI strains) [16], [17], [18]. Our statistical analysis revealed a highly significant correlation of the presence of PorB_IA_ with disseminated gonococcal infection in different countries (see Data Set S1). Although also other mechanism like serum resistance are of high importance during DGI [19], [20], the PorB_IA_-dependent invasion mechanism is suggested to be highly clinically relevant as a means to initiate invasive gonococcal diseases.

We have previously shown that gonococci engage the *scavenger receptor expressed by endothelial cells* (SREC-I) to invade epithelial cells in a PorB_IA_-dependent manner [21]. Scavenger receptors (SR) represent a heterogenic group of membrane receptors belonging to the pattern recognition receptors. SREC-I, like other scavenger receptors, has been defined as receptor for modified lipoproteins including oxidized and acetylated low-density lipoprotein (LDL) but it recognizes also other proteins like Gp96, calreticulin and heat shock protein 90 (HSP90) that are no lipoproteins. The involvement of SREC-I in PorB_IA_-dependent neisserial host cell invasion is thus far the only example of a bacterial pathogen exploiting SREC-I. The signaling cascade leading to PorB_IA_-triggered bacterial uptake into epithelial cells involves Rho GTPases and actin but, in contrast to Opa-dependent invasion, not microtubules, acidic sphingomyelinase, myosin light chain kinase, and Src-kinases [14].

Very little is known about the cellular signaling underlying the switch from local to disseminated infection. Our recent data demonstrate that Vav2- and RhoA-dependent accumulation of actin at membrane rafts actively block invasion of piliated gonococci [22]. Here, we investigated the mechanism of the clinically important SREC-I/PorB_IA_-dependent invasion of gonococci under low phosphate conditions. To our surprise, the signaling that initiates invasion and anti-invasion of gonococci expressing PorB_IA_ and pili both depend on the formation of membrane rafts and caveolin-1 phosphorylation. We identified the p85 regulatory subunit of PI3K/Akt as a new and critical interaction partner of phosphorylated caveolin-1 that is recruited to membrane rafts in a SREC-I-dependent manner during PorB_IA_-dependent invasion. This interaction leads to the activation of a novel bacterial invasion pathway involving the serine threonine kinase D1 (PKD1/PKCμ). Thus, components identified in the present study might aid in identification of novel drug targets for invasive gonococcal diseases.

## Results

### Cytoplasmic domain of SREC-I is not required for gonococcal invasion

**Figure 1:**
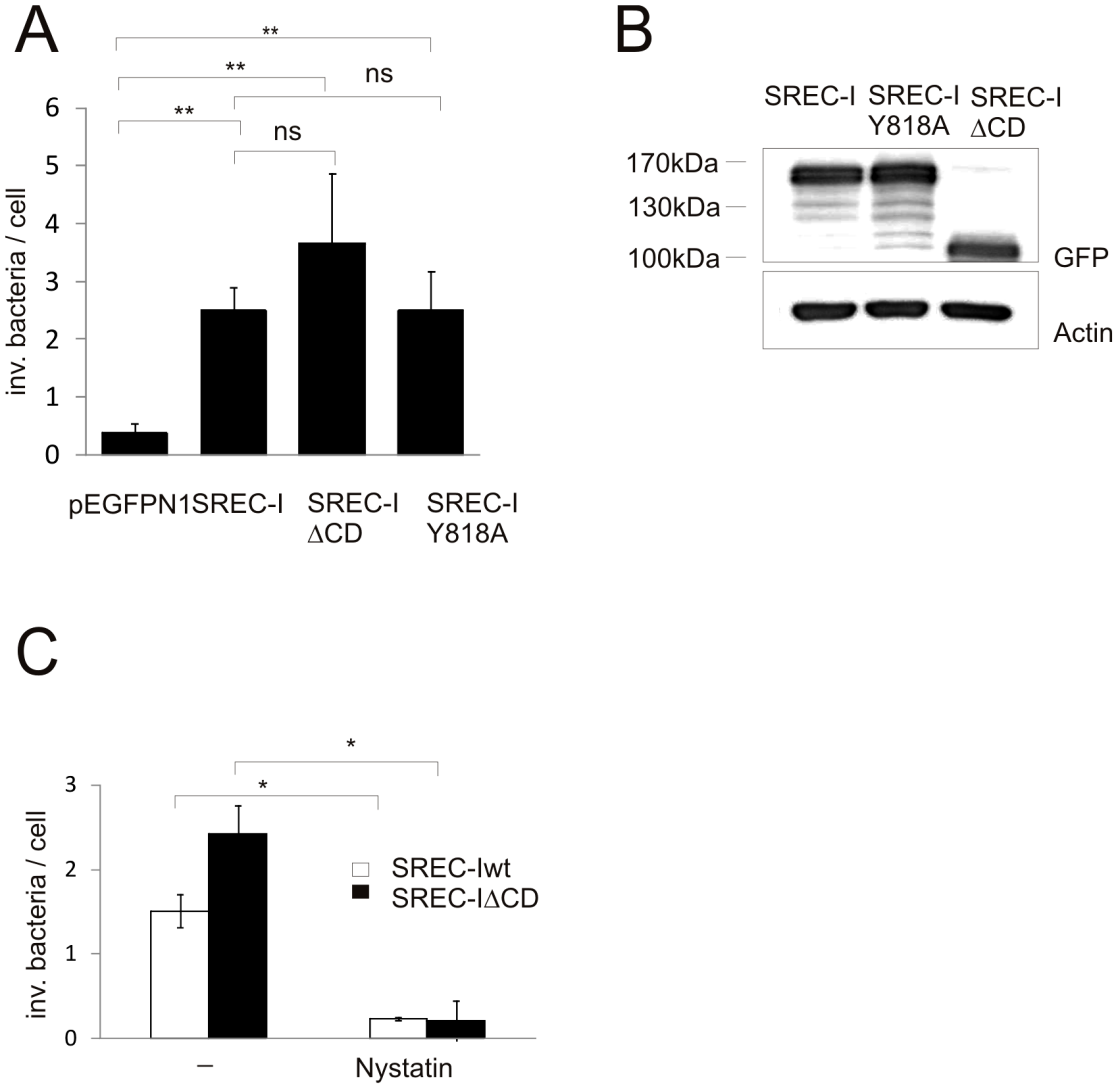
Deletion of the SREC-I cytoplasmic domain does not prevent gonococcal uptake. CHO cells were transfected with pEGFPN1 (control) and constructs overexpressing wildtype SREC-I-GFP (SREC-I), SREC-IY818A-GFP (SREC-IY818A), and SREC-I-GFP lacking the cytoplasmic domain (SREC-IΔCD). (**A**) 24 h post transfection these cells were infected with N927 (PorB_IA_, P^−^) at a MOI of 50. Invasive bacteria were enumerated by confocal microscopy from 50 randomly chosen cells using differential immunostaining. (**B**) Whole-cell lysates were analyzed by western blotting using an anti-GFP and anti-Actin antibody. (**C**) CHO cells transfected with plasmid encoding SREC-I (white bars) or SREC-IΔCD (black bars) were either left untreated (-) or treated with 25 µg/ml Nystatin for 1 h and infected with N927 (PorB_IA_, P^−^) at a MOI of 50. Invasive bacteria were counted from 50 randomly chosen cells using differential immunostaining. The graphs show the mean ± SD of two independent experiments. p<0.05: *; p<0.01: **.

We previously demonstrated that PorB_IA_ mediates invasion of gonococci independent of Opa adhesins and T4P by interacting with the SREC-I receptor [14], [21]. Expression of SREC-I in naturally SREC-I-deficient Chinese hamster ovary (CHO) cells reconstitutes invasion of strain MS11 N927 (PorB_IA_) under low-phosphate conditions, whereas gonococcal strains expressing PorB_IB_ are not internalized. Since internalization signals for scavenger receptors have not been identified so far, we speculated that the 388 amino acids long SREC-I cytoplasmic domain (CD) and phosphorylation of amino acid residues might play a role in signal transduction processes leading to the engulfment of PorB_IA_-expressing gonococci. Because kinase inhibitor studies revealed a role of the tyrosine kinase Abl1 in PorB_IA_-dependent invasion (see below), we first searched for relevant motifs in the CD domain of SREC-I. A computational analysis using NetworkKIN (http://networkin.info) identified Tyr818 (consensus sequence R*XX*E*XX*Y^818^) as a potential Abl1 phosphorylation site. We generated different SREC-I expression constructs fused to GFP (SREC-I-GFP) for the transient expression in CHO cells. Surface exposition of SREC-I wt and truncated SREC-I constructs was similar as demonstrated by FACS-analysis (Fig. S1A). Intracellular bacteria associated with the transfected cells were then quantified by immunofluorescence staining. A Y818A mutant of SREC-I-GFP was similarly efficient as the wildtype derivative in mediating invasion of strain N927 (PorB_IA_, Opa^−^, P^−^) into CHO cells in the absence of phosphate (Fig. 1A,B), suggesting that Y818 phosphorylation is not required for PorB_IA_-mediated uptake of N927. We then generated a SREC-I mutant lacking the entire cytoplasmic domain of SREC-I ranging from amino acids 453 to 830 (SREC-IΔ_AA453-830_; SRECIΔCD). To our surprise, invasion was not prevented in CHO cells expressing SREC-IΔCD (Fig. 1A,B). Thus we conclude that the cytoplasmic domain of SREC-I is not required for PorB_IA_-mediated uptake of gonococci into epithelial cells.

### SREC-I-mediated gonococcal invasion depends on caveolae

Receptors may transmit extracellular signals independent of their CD by associating with ordered assemblies of proteins and lipids called membrane rafts [23]. To test whether the association with membrane rafts is required for gonococcal invasion, CHO SREC-I and CHO SREC-IΔCD transfected cells were treated with the membrane raft-disrupting agent nystatin before infection with N927 (PorB_IA_, P^−^, Opa^−^). Nystatin pretreatment led to a drastic (Fig. 1C) and dose-dependent (Fig. S2B) reduction of invasion compared to untreated control cells. These results were confirmed in Chang conjunctiva cells (Fig. S2A). A similar strong inhibition of invasion could be achieved by depleting cholesterol from host cell membranes with methyl-β-cyclodextrin (MβCD, Fig. S2D). Washout of MβCD and recovery of cholesterol [24] restored invasion of N927 (PorB_IA_, P^−^, Opa^−^). Neither Nystatin nor MβCD affected bacterial (not shown) or cell viability (Fig. S7) or SREC-I surface exposure (Fig. S8). Also, SREC-I co-localised with gonococci in infected Chang cells whereas disruption of lipid microdomains by nystatin treatment interfered with gonococci-SREC-I co-localisation (Fig. S2C) despite an increased surface exposure of SREC-I (Fig. S8). These data together demonstrated a role of membrane rafts for the uptake of PorB_IA_-expressing gonococci via SREC-I.

**Figure 2:**
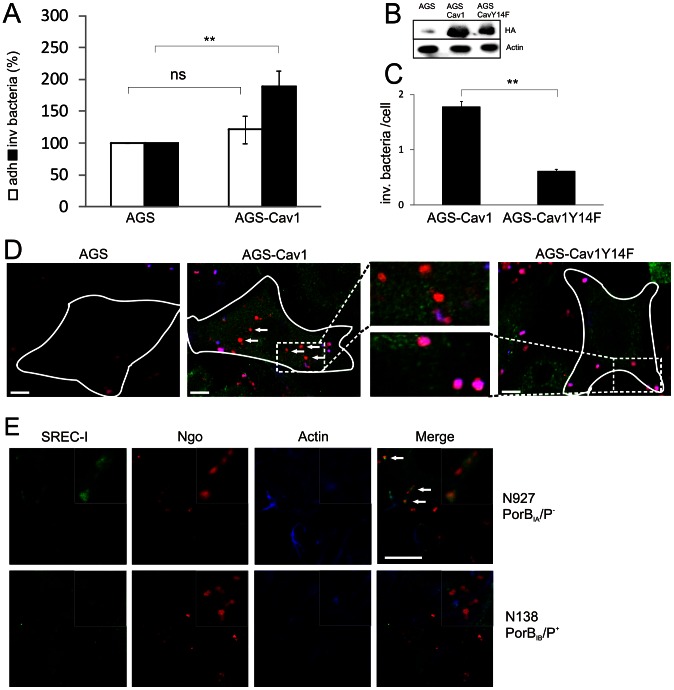
Caveolin is required for N927 invasion. (**a**) AGS cells (AGS) and a transgenic AGS cell line expressing caveolin-1 (AGS Cav1wt) were infected with N927 (PorB_IA_, P^−^) at MOI 50. Adherent (adh.) and intracellular (inv.) bacteria were quantified by gentamicin protection assays. The number of adherent and invasive bacteria of AGS cells was taken as 100%. The graph shows mean values ± SD of three independent experiments done in duplicates. White bars: adherent bacteria; black bars: intracellular bacteria p<0.01: ** (**b**) AGS cell expressing an HA-tagged wt (AGS Cav1wt) or mutant (AGS Cav1Y14F) caveolin were analyzed by Western blot using an HA antibody. (**c**) Intracellular N927 (PorB_IA_, P^−^) of the experiment shown in figure (D) were quantified by differential immunofluorescence. p<0.01: **. (**D**) AGS cells were transiently transfected with HA-tagged Cav1 (AGS-Cav1) or Cav1Y14F (AGS-Cav1Y14F) and infected with N927 at MOI 25. Adherent (pink) and intracellular (red; white arrows) bacteria were detected by differential immunofluorescence. Caveolin expression was visualized with an HA antiserum and a Cy2-conjugated secondary antibody (green). Scale bar: 10 µm (**E**) SREC-I is recruited to N927 (PorB_IA_, P^−^)(white arrows), but not N138 (PorB_IB_, P^+^). Chang cells were infected with SNARF-labeled bacteria at an MOI 25. SREC-I was detected with a polyclonal serum against SREC-I and a Cy2-conjugated secondary antibody. Co-localisation of SREC-I and gonococci was analyzed by confocal fluorescence microscopy. Scale bar: 10 µm.

Membrane raft-dependent internalization of cargo is frequently associated with caveolae, variably sized pits that form in the plasma membrane and are enriched in the major structural protein caveolin-1 (Cav1) [25]. To elucidate the involvement of Cav1 in the uptake of N927, the Cav1-negative gastric cancer cell line AGS and a corresponding Cav1-expressing AGS transgenic line (AGS-Cav1) were infected with N927 (PorB_IA_, Opa^−^, P^−^). Whereas N927 failed to efficiently invade AGS cells, we detected gonococci in AGS-Cav1 cells (Fig. 2A), suggesting that Cav1 is involved in uptake of PorB_IA_ gonococci. Caveolin phosphorylation at tyrosine 14 has previously been linked to raft internalization [26]. We therefore examined whether phosphorylation of Cav1 at Tyr14 is required for invasion via SREC receptors by the expression of the phosphorylation defective mutant Cav1-Y14F in AGS cells (Fig. 2B). Uptake of N927 was inhibited in AGS cells expressing Cav1-Y14F (Fig. 2C, 2D, S3A), demonstrating an important role for phosphorylation at Tyr14 for SREC-I-dependent invasion.

An involvement of Cav1 in gonococcal invasion was unexpected since we recently showed that Cav1 is required to prevent uptake of piliated gonococci into epithelial cells [22]. In this case, Cav1 is recruited to attachment sites of piliated PorB_IB_ gonococci, phosphorylated at Tyr14 and interacts with Vav2 and its substrate the small GTPase RhoA, which then leads to the establishment of stress fibers beneath the microcolonies thereby impeding bacterial uptake [22]. The finding that PorB_IA_ or PorB_IB_/P^+^ gonococci in an isogenic background either induce or prevent their uptake via Cav1-dependent mechanism thus illustrates specific differences in Cav1-mediated signaling of both processes.

In contrast to pilus-mediated adherence PorB_IA_-triggered invasion depends on a low-phosphate environment. Therefore we tested the influence of the invasion medium on the infection outcome and whether non-piliated PorB_IA_-expressing or piliated PorB_IB_-expressing gonococci interact with SREC-I under low phosphate conditions. Chang cells were infected with either N927 (PorB_IA_, P^−^) or N138 (PorB_IB_, P^+^), an isogenic strain possessing PorB_IB_ instead of the SREC-I-interacting PorB_IA_ porin. In SREC-I immunofluorescence studies SREC-I frequently co-localized with N927 (43%), but rarely with N138 (7%) (Fig. 2E), suggesting that SREC-I recruitment is exclusive for PorB_IA_-expressing strains. By gentamicin protection assays we compared the invasion efficiencies of either strain under phosphate free conditions. Contrary to N927, N138 failed to invade Chang cells (Fig. S3D). Further, we observed the formation of actin aggregates (Fig. S3B) and bacterial microcolonies (Fig. S3C) for N138 infection but not for N927. This strongly suggests that not the low-phosphate conditions but rather the specific interaction with the SREC-I receptor trigger the uptake of gonococci.

### N927 invasion depends on Cav1-interacting proteins Abl1 and PLCγ1

**Figure 3:**
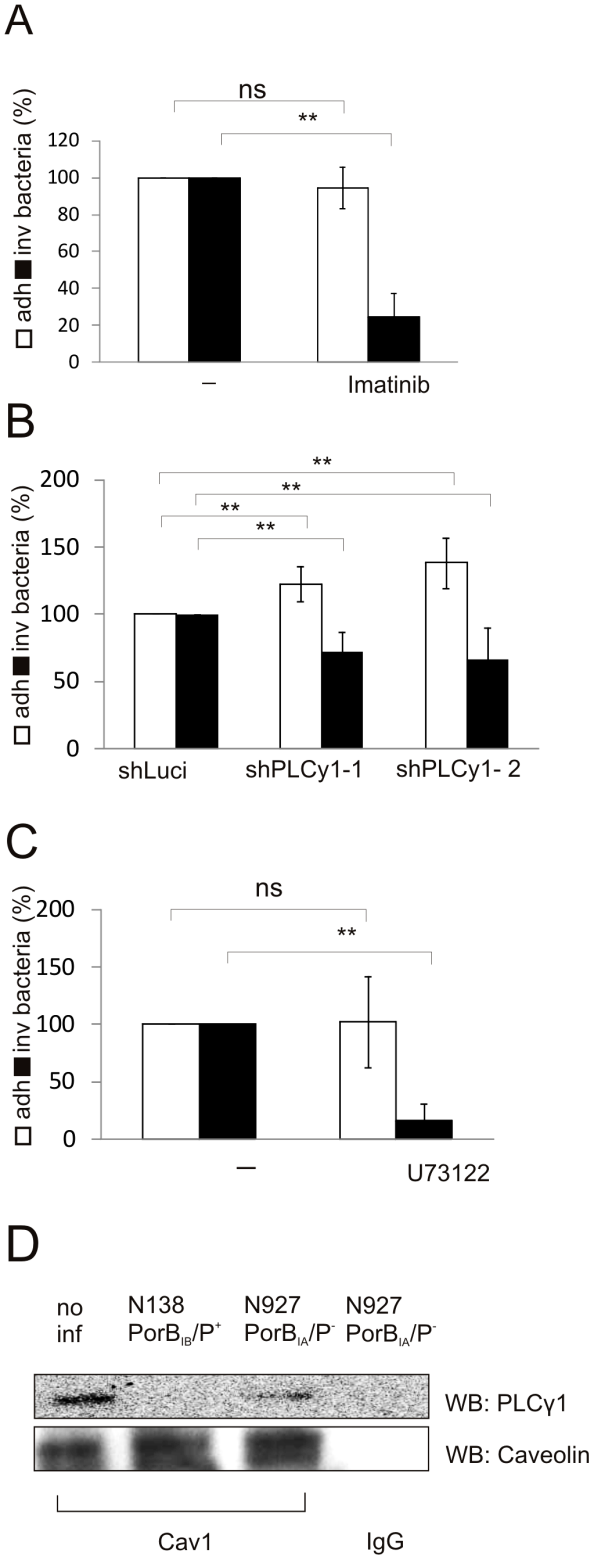
Abl1 and PLCγ1 are essential for N927 invasion. (**A**) Chang cells were pretreated for 1 h with the Abl1 inhibitor Imatinib (10 µM) and subsequently infected with N927 (PorB_IA_, P^−^) at an MOI 10 for 30 min. Adherence (white bars) and invasion (black bars) were quantified by gentamicin protection assays. The number of adherent (adh.) and invasive (inv.) bacteria of untreated control cells was set to 100%. The bar chart shows mean percentages ± SD of three independent experiments each performed in duplicate. (**B**) shRNA-mediated downregulation of PLCγ1 in Hela cells results in decreased internalization of N927 (PorB_IA_, P^−^). Control cells (shLuci) as well as shPLCγ1 cells (shPLCy1-1, shPLCy1-2) were infected with strain N927 (MOI 10, 30 min) and adherence (white bars) as well as invasion (black bars) were analyzed by gentamicin protection assays. The numbers of adherent and invasive bacteria of control cells (shLuci) were set to 100%. Shown are mean percentages ± SD of three independent experiments performed in duplicate. (**C**) Chang cells were either left untreated (-) or pretreated for 30 min with PLCγ1 inhibitor U73122 (10 µM) and infected with N927 (MOI 10, 30 min). Adherence (white bars) and invasion (black bars) were quantified by gentamicin protection assays. The numbers of adherent (adh.) and invasive (inv.) bacteria of untreated control cells were set to 100%. The graph shows mean values ± SD of three independent experiments performed in duplicates. (**D**) PLCγ1 co-precipitates with Cav1 in N927-infected cells and untreated cells. Chang cells were infected with either N927 (PorB_IA_,P^−^) or N138 (PorB_IB_,P^+^) MOI 20 for 1 h. Endogenous Cav1 was precipitated from infected and not infected control (no inf) cells and co-precipitated PLCγ1 was detected by Western blot. p<0.01: **.

Recently, we identified several Cav1 pTyr14 interacting proteins, by screening microarrays of recombinant human SH2 and other phosphotyrosine binding (PTB) domains. High affinity interaction partners included Abl family kinases as well as phospholipase Cγ1 (PLCγ1) and Vav2 [22]. Of these, only Vav2 turned out to play a major role in pilus-mediated inhibition of gonococcal invasion [22]. We therefore tested if either Abl1 or PLCγ1 are required for the invasion of PorB_IA_ gonococci (N927; PorB_IA_, P^−^). Imatinib, a selective inhibitor for the tyrosine kinase Abl1, reduced internalization of N927 by more than 70% (Fig. 3A) whereas adherence was not affected suggesting that Abl1 is required for invasion. To test whether PLCγ1 is involved in N927 invasion, several PLCγ1 knock-down cell lines were tested for their ability to engulf N927 bacteria. Invasion was reduced in all these cell lines (Fig. 3B, S4A). Furthermore the PLCγ1 inhibitor U73122 efficiently prevented the uptake of N927, corroborating a role of PLCγ1 activity in the signaling cascade leading to uptake of gonococci via SREC-I (Fig. 3C).

We then tested whether PLCγ1 specifically interacts with Cav1 in cells infected with N927. Pull-down experiments of endogenous proteins demonstrated that PLCγ1 interacted with Cav1 in cells either infected with N927 and non-infected cells, but not in cells infected with N138 (Fig. 3D), suggesting that PLCy1 is a constitutive partner in Cav1-containing complexes.

### Identification of Cav1-pY14 interaction partners

**Figure 4:**
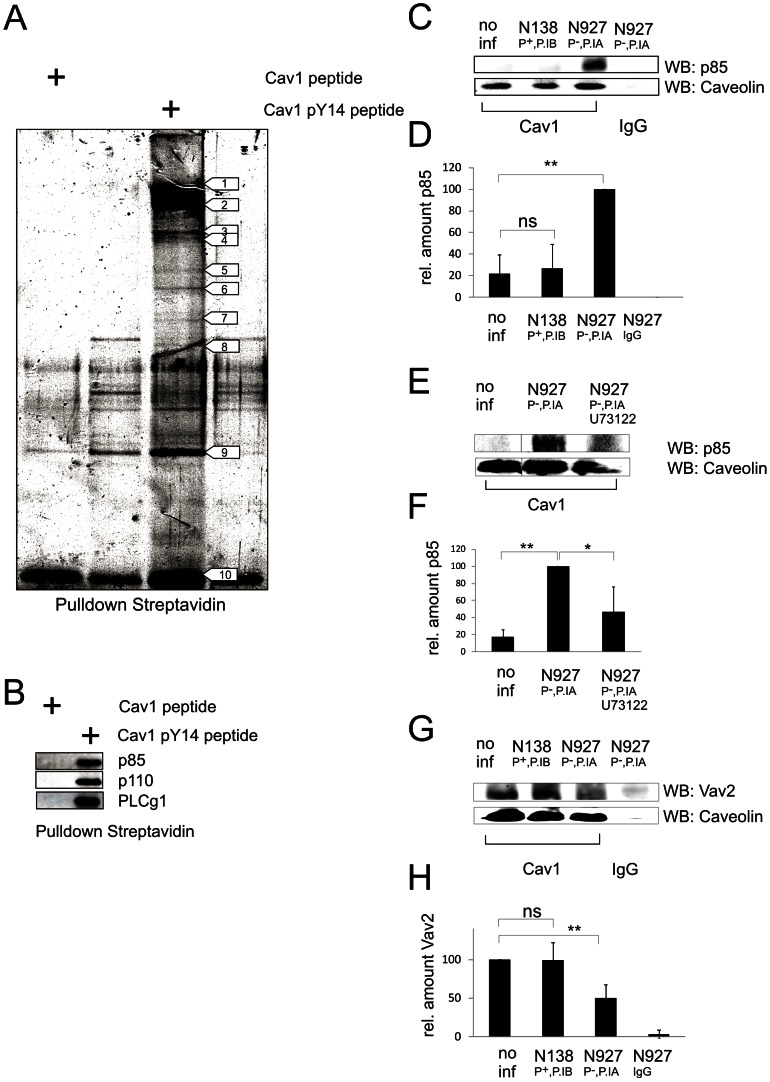
Identification of new Cav1 pTyr14 interaction partners. (**a**) Pull-down of Cav1-pY14 interaction partners. Biotin-labeled Cav1 peptides either with (Cav1 pY14 peptide) or without (Cav1 peptide) phosphorylated Tyr14 were used as baits to precipitate interacting proteins from AGS cell lysates using a streptavidin-agarose pulldown assay. Precipitates were separated by SDS-PAGE and proteins visualized by silver staining. The proteins enriched in the Cav1 pY14 pulldown (indicated by numbers; see also table 1) were identified by Maldi-MS/MS. (**b**) Western blot analysis of proteins precipitated from ME-180 cells using either biotin-labeled phosphorylated or non-phosphorylated Cav1 peptides as bait. PLCy1 as well as the regulatory p85 and catalytic p110 subunit of PI3K, are only detected in precipitates of the phosphorylated peptide with the respective primary antibodies. (**c**) PI3K-p85 interacts with Cav1 in N927 infected cells. Chang cells were infected with either N927 (PorB_IA_, P^−^) or N138 (PorB_IB_, P^+^) at an MOI 20 for 1 h or not infected (no inf). Endogenous Cav1 was precipitated and co-precipitated proteins were detected by Western blot with antibodies specific for Cav1 or PI3K-p85. (**D**) Relative amount of PI3 kinase quantified from (C). The bars represent the mean values ± SD of three independent experiments. (**E**) PI3 kinase recruitment to caveolin depends on activity of PLCy1. The experiment was performed as described in (C). 30 min prior to infection the PLCy1 inhibitor U73122 (10 µM) was added to the cells. (**F**) Relative amount of p85 quantified from three independent experiments. Shown are the mean values ± SD. (**G**) Interaction of Cav1 with Vav2 tested under conditions as described in (C). (**H**) Relative amount of Vav2 quantified from the experiment shown in (G). The bar graph shows the mean value of three independent experiments ± SD. p<0.05: *; p<0.01: **.

So far our data demonstrated that Abl1 and PLCγ1 are required for PorB_IA_-dependent invasion whereas at least PLCγ1 is dispensable for pili-induced prevention of bacterial uptake [22]. Since PLCγ1 seemed to be part of a constitutive protein complex with Cav1 we reasoned that an unknown host factor determines the specific fate of N927 (PorB_IA_, P^−^) and N138 (PorB_IB_, P^+^). Therefore, a biochemical assay was developed to identify native Cav1-pY14 signaling partners (Text S1). Biotin-labeled, phosphorylated or non-phosphorylated Cav1 peptides comprising amino acid residues 7–21 of Cav1 were used as bait in pull-down assays. Interacting proteins from Cav1-negative AGS cells were captured and subsequently analyzed by Maldi-MS/MS (Fig. 4A; numbered arrows; Table 1). Most interestingly, p85, the regulatory subunit of the phosphoinositide 3-kinase (PI3K-p85), was identified as a novel interaction partner of the Cav1-pY14 phosphopeptide (Figure 4A, arrow No. 7). Additionally, myosin IB, myosin ID, non-muscle myosin heavy chain IIA, cytokeratin 1, β-actin and splicing factor proline/glutamine rich (SFPQ/PSF) were identified as possible Cav1-pY14 binding partners (Fig. 4A numbered arrows and Table 1).

**Table 1:** Phospho-Tyr14-Cav1 binding partners identified by MALDI-TOF/TOF after streptavidin pulldown of biotin-labeled Tyr14-Cav1 peptides.

Band No.:	Identified protein:	Mascot Score	Sequence coverage (%)	NCBI Protein Database No.	NCBI Gene ID
1,2,3,4,5,7	non muscle myosin heavy chain IIA	260,455,155142,81,46	25,28,18,19,14,9	12667788	4627
5	myosin IB	45	13	44889481	4430
6	splicing factor proline/glutamine rich (SFPQ/PSF)	168	25	23956214	6421
6	myosin ID	59	15	119600629	4642
7	PI3K, regulatory subunit p85α	75	17	32455248	5295
8	cytokeratin 1	242	31	11935049	3848
9	β-actin	345	61	15277503	60
10	Chain A, Streptavidin Mutant	332	38	34811425	-

Binding of PI3K-p85 and PLCγ1 to biotinylated Cav1-pY14 peptides was confirmed by streptavidin-agarose pull-down-assays from cell lysates and Western blot detection. PLCγ1, p85 and also p110, the catalytic subunit of PI3K, bound exclusively to the phosphorylated Cav1 peptide (Fig. 4B), confirming these proteins as targets of phosphorylated Cav1.

### PI3K is required for invasion of disseminated gonococci

To investigate the physiological relevance of PI3K for invasion of N927 (PorB_IA_, P^−^), endogenous Cav1 was immunoprecipitated from infected and non-infected cells and co-immunoprecipitation of PI3K-p85 was demonstrated by Western blot analysis. Interestingly, a strong interaction was demonstrated in N927, but not in N138 (PorB_IB_, P^−^) infected cells (Fig. 4C,D). Interaction of Cav1 depended on PLCγ1 activity indicating that lipid second messengers generated by PLCγ1 are involved in PI3K-p85 recruitment to Cav1 (Fig. 4 E,F). Pilus-mediated invasion inhibition depends on the interaction of Vav2 with Cav1-pY14 leading to RhoA activation and actin accumulation below the gonococcal microcolony [22]. In cells infected with N927 (PorB_IA_, P^−^), however, less (50%) Vav2 was present in endogenous caveolin-1 complexes than in cells infected with N138 (99%, PorB_IB_, P^+^) or even non-infected cells (100%, Fig. 4 G,H). Small amounts of Vav2 in pulldowns of Cav1 from the cells infected with N927 (PorB_IA_, P^−^) may thus originate from a small population of non-infected cells since not all cells are infected under the used conditions. A role of Vav2 for PorB-mediated invasion can unambiguously be excluded, since suppression of Vav2 expression in HeLa cells had no effect on invasion of N927 (PorB_IA_, P^−^) (Fig. S4B,C). These data indicate that the displacement of Vav2 and the recruitment of PI3K-p85 shift the signaling cascade at the level of Cav1 from invasion inhibition to invasion.

**Figure 5:**
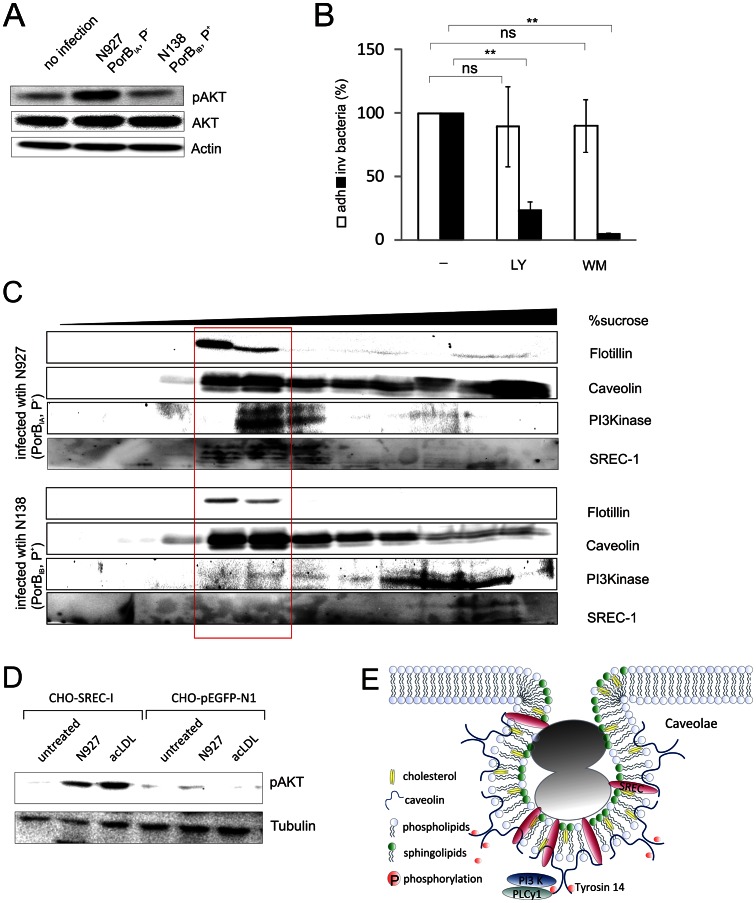
PI3 kinase is required for N927 invasion and is recruited to caveolae. (**A**) Activation of PI3K shown by phosphorylation of Akt. Whole cell lysates of Chang cells infected with either N927 (PorB_IA_, P^−^) or N138 (PorB_IB_, P^+^) at an MOI 50 for 30 min were subjected to SDS PAGE and Western blot using anti-phospho-Akt, anti-Akt and anti-Actin antibodies. (**B**) Chang cells were pretreated for 1 h with PI3K inhibitors LY294002 (LY, 10 µM) or Wortmannin (WM, 1 µM) and infected with N927 (MOI 10, 30 min). Adherence (white bars) and invasion (black bars) were quantified by gentamicin protection assay. The number of adherent and invasive bacteria of untreated control cells was set as 100%. The graph shows mean values ± SD of three independent experiments performed in duplicates. p<0.01: ** (**C**) Distribution of signaling molecules in membrane rafts of infected cells. Chang cells were subjected to subcellular fractionation after infection either with N927 (PorB_IA_, P^−^) or N138 (PorB_IB_, P^+^) (MOI 20) for 1 h (see Text S1). Flotillin was detected as marker for the membrane raft fraction (fraction 4–5) separated from most cellular proteins (fraction 8–12). Caveolin, SREC-I (multiple bands represent differentially glycosylated forms), Flotillin and PI3K, were detected by Western blot analysis. (**D**) CHO cells stably transfected with either SREC-I WT (CHO-SREC-I) or empty vector control (CHO-pEGFP-N1) were infected with N927 (PorB_IA_, P^−^) at an MOI of 50 or treated with 2 µg/ml acLDL (Invitrogen) for 5 min. Whole cell lysates were subjected to SDS-PAGE and Western blot analysis using anti-phospho-Akt and anti-Tubulin antibodies. (**E**) Graphical representation of SREC-I gonococci interaction in Caveolae. Upon infection with N927 SREC-I localizes in cholesterol, sphingolipid and caveolin-1 rich membrane rafts. PLCy1 and PI3K are recruited to phosphorylated Cav1 (Tyrosin 14) and initiate the signaling cascade leading to endocytic uptake of the gonococci. Adapted from [25].

Recruitment of PI3K-p85 upon infection with N927, but not with the isogenic strain N138 induced the activation of the kinase, as determined by Western blot analysis with an antibody that detects the active phosphorylated form of Akt (Fig. 5A), a downstream target of PI3K. Activation of PI3K also depended on the presence of Cav1 since Akt phosphorylation was detected in AGS-Cav1 upon N927 infection, but not in AGS cells deficient in Cav-1 expression. In line with the selective recruitment of PI3K-p85 to Cav1 upon N927 infection, infection of AGS-Cav1 cells with N138 did not increase PI3K activity (Fig. S5A,B). In addition PI3K was not activated upon infection with either N313 (PorB_IB_, Opa_57_), a gonococcal strain interacting with all CEACAM receptors [27], or N931 (PorB_IB_, Opa_50_), which interacts with the HSPG receptor [8], [9] (Fig. S5C,D). This demonstrated that PI3K invasion signaling occurs specifically in PorB_IA_-expressing gonococcal infections.

Inhibitor studies were performed to test whether PI3K is required for PorB_IA_-dependent invasion. The number of internalized bacteria was decreased in presence of the specific PI3K inhibitors LY294002 and wortmannin, whereas bacterial adherence was unchanged (Fig. 5B), demonstrating a crucial role of PI3K for invasion of N927. The specific activation of PI3K upon infection with N927 (PorB_IA_, P^−^), but not with N138 (PorB_IB_, P^+^) as well as the requirement of PI3K activity for N927 invasion were confirmed in End1 cells (Fig. S5E,F), demonstrating the presence of this entry route for PorB_IA_ gonococci in non-transformed cells.

As shown above membrane raft domains are involved in PorB_IA_-triggered invasion processes. Therefore we speculated that bacterial uptake is initiated after accumulation of signaling molecules in these microdomains. We thus investigated whether SREC-I and PI3K are recruited to membrane rafts during infection. By sucrose gradient centrifugation SREC-I and PI3K-p85 were found to be enriched in Cav1- and flotillin-rich fractions of cells infected with N927, but not in cells infected with N138 (Fig. 5C), supporting the requirement of membrane rafts in SREC-I-dependent recruitment of PI3K.

Treatment of CHO cells transfected with SREC-I expression constructs, but not control cells responded with the activation of PI3K either upon infection with PorB_IA_ gonococci or upon stimulation with acLDL (Fig. 5D), a known trigger of SREC-I endocytic uptake [28]. These data demonstrate that stimulation of SREC-I is sufficient to activate PI3K.

### PKD1 as novel mediator of PorB_IA_-dependent invasion

**Figure 6:**
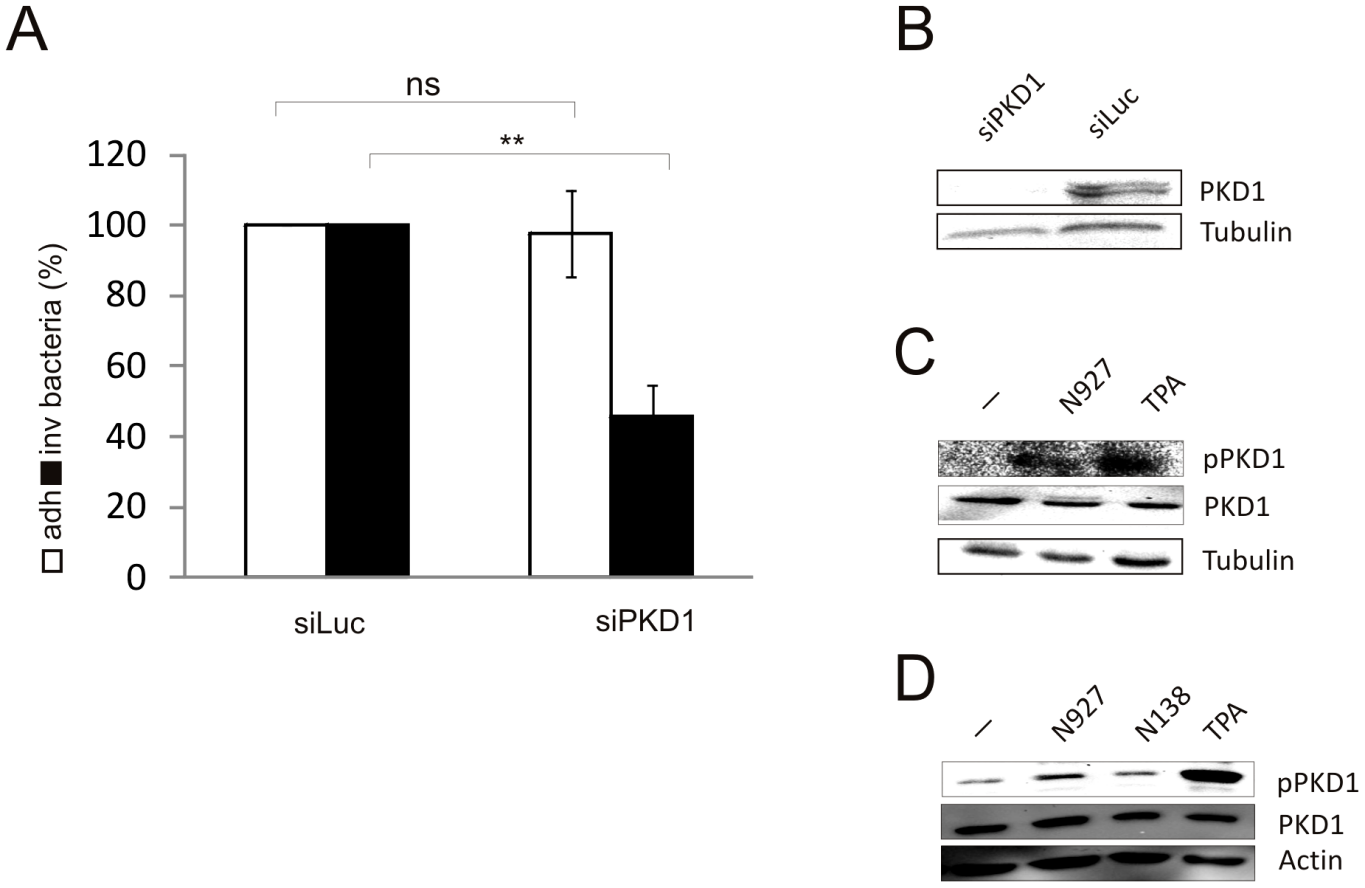
PKD1 is required for invasion of N927. (**A**) Chang cells were transfected with siRNAs against PKD1 (siPKD1) and luciferase (siLuc) as control. The cells were infected with N927 (PorB_IA_, P^−^; MOI 10) for 30 min 72 h after transfection of siRNAs. Intracellular (inv., black bars) and adherent (adh., white bars) bacteria were quantified by gentamicin protection assay and the number of adherent or invasive bacteria of control cells (siLuc) was set to 100%. Shown are the means ± SD of three independent experiments performed in duplicates. p<0.01: ** (**B**) Knock down of PKD1 in Chang cells was verified by Western blotting. β-tubulin was used as loading control. (**C**) Endogenous levels of phosphorylated PKD1 (pPKD1) were assayed after infection with N927 (MOI 100) or treatment with the known activator 12-O-Tetradecanoylphorbol 13-acetate (TPA, 0.2 µM) for 30 min. Whole cell lysates were analyzed by immunoblotting using phospho-specific PKD1 antibody (detects pSer744 and pSer748). (**D**) PKD1 was overexpressed by transfection of PKD-HA expression construct (Addgene; [41]) in Chang cells and phosphorylation was detected as described in (C).

The function of PLCγ1 in SREC-mediated invasion of N927 (PorB_IA_, P^−^) suggested the involvement protein kinase C family members (PKC), since PLCγ1 generates lipid second messengers activating certain PKCs. PKCs are classified as conventional (α, β1, β2, γ), novel (δ, ε, η, θ, μ), and atypical (ζ, λ) isozymes. As inhibitor studies indicated a role for PKD1 (PKCμ) in PorB_IA_/SREC-I based invasion (not shown), we conducted siRNA experiments thereby selectively knocking down PKD1. Invasion was reduced by approx. 60% in PKD1 knock-down Chang cells when compared to cells treated with a control siRNA (Fig. 6A,B). PKD1 activation is dependent on the phosphorylation of two activation loop sites at Ser744 and Ser748 [29]. As demonstrated with Western Blots using phospho-specific antibodies infection with N927 but not with N138 activated PKD1 (Fig. 6C,D). This indicates that the activation of PKD1 is exclusive for PorB_IA_-gonococci infection.

### The PorB_IA_/SREC-I invasion signaling pathway

**Figure 7:**
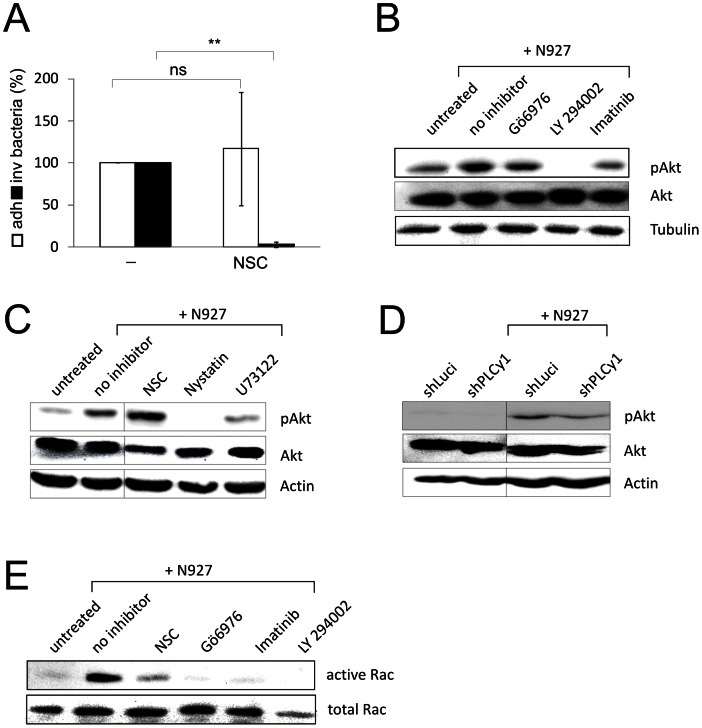
Signaling cascades leading to Akt phosphorylation and Rac1 activation. (**A**) Involvement of Rac1 in N927 (PorB_IA_, P^−^) invasion. Chang cells were pretreated for 1 h with the Rac1 inhibitor NSC23766 and subsequently infected with N927 (MOI 10; 30 min). Adherence (white bars) and invasion (black bars) were quantified by gentamicin protection assay. Shown are the mean values ± SD of three independent experiments performed in duplicates. The number of adherent (adh.) and intracellular (inv.) bacteria recovered from untreated control cells was set to 100%. p<0.01: **. (**B,C**) Chang cells were pretreated with kinase inhibitors (B) or inhibitors for other signaling factors (C) for 1 h, infected with N927 (PorB_IA_, P^−^) at MOI 50 for 30 min. Whole cell lysates were separated by SDS-PAGE and transferred to PVDF membranes. Blots were probed with antibodies against pAKT, as a readout for PI3K activity, AKT and either β-tubulin or actin as loading control. Inhibitors: Gö6976 for PKD1, LY294002 for PI3K, Imatinib for Abl1, NSC for Rac1, Nystatin for membrane rafts, U73122 for PLCy1. (**D**) PI3K is localized downstream of PLCγ1. Control cells (shLuci) as well as shPLCγ1 cells were infected with N927 (MOI 50; 30 min) and PI3K activity was assessed by detection of Akt phosphorylation as described in (B). (**E**) A novel signaling cascade leading to Rac activation after infection with N927. Chang cells were pretreated with the individual inhibitors for 1 h and infected with N927 at MOI 50 for 30 min. Active Rac1 was precipitated from cell lysates as described in Experimental Procedures. Western Blots were developed with an anti-Rac antibody.

We have previously shown that a hitherto unidentified Rho GTPase family member is required for PorB_IA_-mediated invasion [14]. Here, we made use of NSC23766, a cell-permeable pyrimidine compound that specifically inhibits Rac1, without affecting Cdc42 and RhoA activation [30]. Inhibition of Rac1 in Chang epithelial cells completely blocked invasion of N927 (PorB_IA_, P^−^), whereas adherence was unaltered as was determined by gentamicin protection assays (Fig. 7A) and differential immunostaining (not shown). Hence Rac1 is the Rho GTPase involved in low phosphate dependent invasion.

To delineate the hierarchy of the PorB_IA_/SREC-I invasion signaling pathway, we applied inhibitors of the identified components and assayed the activation of PI3K via Akt phosphorylation as well as Rac1 upon infection with N927. Inhibition of Abl1 prevented Akt phosphorylation. This is in line with Abl1 being located upstream of PI3K in this signaling pathway. The pAkt signal completely disappeared after treatment with the PI3K inhibitor LY294002 (Fig. 7B). Treatment of host cells with the PKD1 inhibitor, Gö6976, had no effect on the infection-induced activation of PI3K, placing PKD1 downstream of PI3K (Fig. 7B). Interestingly, PLCγ1 activity (Fig. 7C,D) was required for Akt phosphorylation, whereas even basal levels of active PI3K disappeared upon destruction of membrane rafts by Nystatin (Fig. 7C). Since the inhibition of Rac1 by NSC failed to interfere with PI3K activation (Fig. 7C) and activation of Rac1 was prevented by all other inhibitors (Fig. 7E), Rac1 must be localized at the end of the investigated signaling cascade. In its entirety, PorB_IA_/SREC-I-dependent gonococcal invasion in epithelial cells thus constitutes a novel invasion pathway involving caveolae (Cav1), Abl1 kinase, PLCγ1, PI3K, PKD1 and Rac1.

### Pili interfere with PorB_IA_-dependent invasion

**Figure 8:**
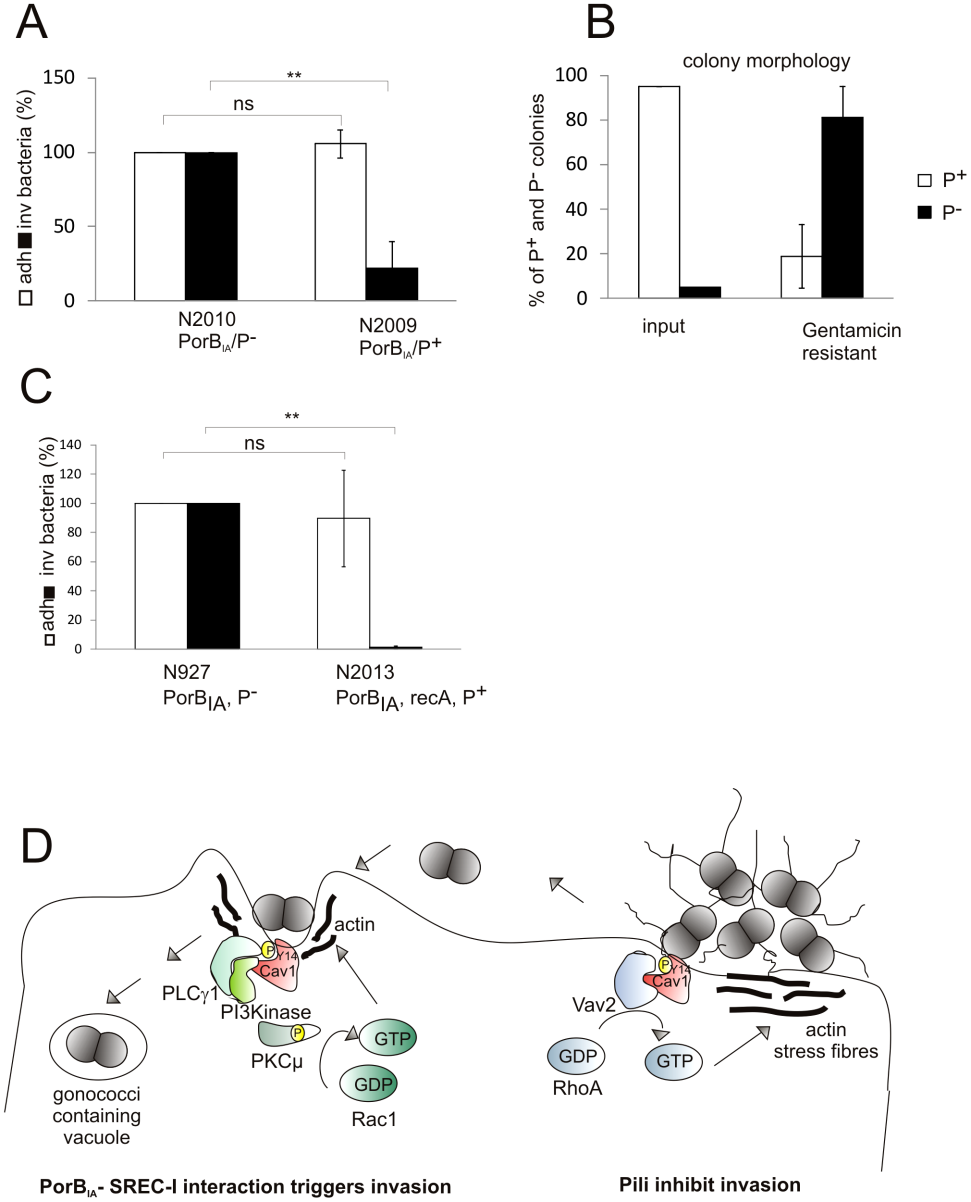
Pili interfere with PorB_IA_-dependent invasion. (**A**) Chang cells were infected with N2009 (PorB_IA_, P^+^) or N2010 (PorB_IA_, P^−^) at MOI 10 for 1 h and then analyzed by gentamicin protection assays. The number of adherent (white bars) and invasive (black bars) bacteria of the strain N2010 was taken as 100%. The graph shows mean values ± SD of two independent experiments performed in duplicates. (**B**) Intracellular N2009 are highly enriched for non-piliated variants. Piliated (white bars, Pili+) and non-piliated (black bars, Pili−) gentamicin resistant N2009 gonococci recovered from the experiment shown in (A) were quantified for their pilus phenotype with a steromicroscope. (**C**) Chang cells were infected at an MOI of 10 for 30 min with N927 (PorB_IA_, P^−^) or with N2013 (*recA* ^i^, PorB_IA_, P^+^), a *rec*A mutant which cannot undergo *pil*E recombination. The bar chart shows the results of a gentamicin protection assay as the mean values ± SD of three independent experiments done in duplicates. The number of adherent (white bars) and invasive (black bars) bacteria of N927 was set to 100%. p<0.01: ** (**D**) Model of the molecular events underlying the switch from local to invasive gonococcal infection.

The role of pilus phase variation during invasion and transcytosis of epithelial cells is still controversial [31], [32]. Since pilus-mediated prevention of invasion and PorB_IA_-triggered invasion were dependent on highly similar signaling pathways, we asked whether PorB_IA_ expression could override the anti-invasive signaling by pili and thus permit invasion of piliated bacteria into epithelial cells. We therefore generated N2009 (P^+^, PorB_IA_) in strain MS11 and N2010 (P^−^, PorB_IA_), a non-piliated derivative of N2009 and performed gentamicin protection assays under low phosphate conditions. Whereas adherence was similar between the piliated and non-piliated derivative under the chosen condition, invasion was reduced about 4-fold for the piliated strain N2009 (Fig. 8A). Since pilus expression is phase variable, we monitored the pilus phenotype of the input strain and bacteria recovered after the gentamicin protection assay. Less than 5% of the colonies of the inoculum were non-piliated when viewed under a stereomicroscope or analyzed by electron microscopy (Fig. S9A). By contrast 80.5% of the recovered bacteria had a P^−^ phenotype (Fig. 8B). We obtained a similar result with the immortalized endocervical cell line End1, supporting a role of pili in the switch from an adherent to an invasive phenotype also in non-transformed cells (Fig. S 9B). Our observation suggested that only non-piliated variants were able to invade host cells and that pilus expression effectively blocked PorB_IA_-dependent invasion. Expression of pili can be frequently switched ‘ON’ and ‘OFF’ by RecA-dependent recombination between silent and expression *pil* loci (frequency about 10^−3^) or by less frequent (frequency about 10^−6^) RecA-independent in- and out-of-frame switches in the pilus assembly gene *pilC*. To test the hypothesis that the non-piliated, invasive variants arise as a consequence of the natural switch-off in pilus expression a *rec*A mutant defective in *pil*E recombination [33] was generated in a piliated PorB_IA_ expressing strain. N2013 (*recA*, P^+^, PorB_IA_) failed to invade Chang cells (Fig. 8C) consistent with a role of pilus phase variation in the transition from an anti-invasive to an invasive phenotype. In contrast, N2015 (*recA*, P^−^, PorB_IA_), a non-piliated *rec*A derivative, invaded into Chang cells (data not shown), ruling out recombination events other than those leading to pilus variation to be involved in PorB-dependent invasion.

## Discussion

The initial interaction of gonococci with their host is mediated by the type 4 pili leading to highly efficient colonization and microcolony formation at the surface of the epithelium of the urogenital tract. We show here that both, pilus-dependent microcolony formation and PorB_IA_-dependent invasion, engage caveosomes although in an antagonistic fashion.

Although the PorB_IA_-triggered invasion requires SREC-I, we were intrigued that the cytoplasmic C-terminal domain of the receptor is dispensable for invasion of strain N927 (PorB_IA_, P^−^). This is reminiscent of endocytosis of modified LDL by SREC-I which is independent of the C-terminal domain [34]. Other receptors such as CEACAM-1 also trigger the uptake of bacteria in the absence of a cytoplasmic domain via membrane rafts [23]. We therefore hypothesize that SREC-I oligomerization following PorB_IA_-dependent binding of *N. gonorrhoeae* is sufficient for receptor recruitment to membrane rafts and initiation of invasion signaling.

In the present work SREC-I was found to be selectively recruited to detergent-resistant microdomains in cells infected with PorB_IA_ invasive gonococci. In line with the localisation of SREC-I to membrane rafts [35], we found that intact membrane rafts and caveolin-1 are required for the uptake of gonococci via PorB_IA_ and SREC-I. Although membrane rafts and caveolin-1 have been implicated in the invasion of bacteria before [36], their role for PorB_IA_ gonococci invasion was unexpected since we previously demonstrated that caveolin-1 aggregation at adhesion sites of piliated gonococci causes a block of invasion [22]. Intriguingly, AGS cells naturally defective for caveolin-1 expression [37] were only efficiently invaded by N927 (exhibiting the PorB_IA_ allele) if caveolin-1 was expressed as a transgene (Fig. 2) whereas isogenic piliated gonococci invaded AGS cells but not the isogenic AGS-Cav1 line [22]. The key question therefore arose how caveolin-1 exerts this dual function as an invasion promoter or an inhibitor.

Since both, invasion promotion (Fig. 2B–D) and inhibition [22], depended on the phosphorylation of caveolin at Tyr14, we first tested Cav-Y14P interacting proteins [22] for a role in strain N927 (PorB_IA_, P^−^) invasion. PLCγ1 turned out to be a constitutive partner of endogenous Cav1 protein complexes in host cells. PLCγ1 activity, however, is required for the uptake of N927 whereas it is not involved in the anti-invasive signaling of piliated gonococci [22]. Interestingly, PLCγ1 activity was required for the recruitment of the p85 regulatory subunit of PI3K identified as a novel caveolin-interacting protein in the present work. Intriguingly, PI3K-p85 was selectively recruited to caveolin-1 complexes in cells infected with strain N927 leading to kinase activation and Akt phosphorylation. By contrast, Vav2 was depleted from these complexes (Fig. 4 G,H). Since the Cav-pY14 interacting exchange factor Vav2 plays a critical role for the invasion block mediated by piliated gonococci [22], our data strongly suggested that the selective recruitment of either Vav2 or PI3K-p85 determines the extra- or intracellular fate of gonococci, respectively.

Using the A431 tumor cell line, Lee et al. have previously demonstrated a role of PI3K for the invasion of piliated gonococci [38]. Since we did not detect significant invasion of piliated gonococci in the two different tumor lines (Chang, ME-180) and the non-transformed End1 cells it is possible that differences in the infection protocol and/or special features of the A431 line account for these contradictory observations.

Additionally, our data clearly demonstrated the involvement of PKD1 in the invasion of N927 (PorB_IA_, P^−^). This is, to our knowledge, the first report on an involvement of this novel PKC in the invasion of pathogenic bacteria. PKD1 has previously been implicated in the control of actin reorganisation and tumor cell migration [39]. Known activators for PKD1 are classical PKCs, phospholipase Cγ, diacylglycerol and PI3 kinase [40]. We ruled out an activation of PKD1 via classical PKCs because Gö6983, a potent inhibitor of these PKCs, did not affect invasion at physiologically relevant concentrations (not shown). It is very likely, that second messengers generated by PLCγ1 and PI3K are critically involved in the activation of PKD1 in the course of N927 infection. There are also indications that Abl1 can activate PKD1, since Abl1 phosphorylates PKD1 at Tyr463. This phosphorylation is known to facilitate the phosphorylation of Ser738/Ser742, which in turn leads to the activation of PKD1 [41].

Activation of PKD1, critical for the uptake of PorB_IA_-expressing gonococci, is not involved in invasion of Opa-expressing bacteria via CEACAM-receptors on Chang cells (Fig. S6A,B) and inhibition of PI3K activity did not prevent invasion via CEACAM3 [42], [43], indicating the usage of alternative infection routes by these phase-variable bacteria. Interestingly, PKD1 knockdown prevented the uptake of Opa_50_-positive gonococci via HSPG receptor (Fig. S6A,B). A not further characterized PKC member has previously been linked to invasion via HSPG [44]. Thus, it is likely that PKD1 represents this protein kinase. Apart from the activity of PKD1 we ruled out further similarities between the SREC-I- and HSPG-mediated uptake processes [14].

Several studies have shown that formation of pili is necessary for efficient adherence of gonococci [4], [7] and that PorB_IA_-expression is associated with serious systemic infections [17], [18]. Our finding that the absence of pili in otherwise isogenic backgrounds is decisive for invasion thus has important implications for the development of invasive gonococcal disease. A pilus-dependent mechanism has recently been proposed for dissemination of *N. meningitidis*. In this case posttranslational modification of pilin determines whether meningococci colonize or invade host tissue [45]. Based on our data we propose that piliated gonococci expressing the PorB_IA_ allele remain extracellular by stabilizing a caveolin-pY14-Vav2 complex followed by the activation of RhoA and the formation of actin aggregates underneath gonococcal microcolonies. Natural pilus phase variation leads to the formation of non-piliated variants. Loss of piliation favors the displacement of Vav2 and the recruitment of PI3K-p85 to the caveolin-pY14 in a SREC-I and PLCγ1-dependent manner which overrides the invasion inhibition. As a consequence, PI3K is activated and a unique signaling pathway leading to the activation of PKD1 and Rac1-dependent invasion is initiated. The concept emerging from our data suggests a role of lipid microdomains as signaling platforms for invasion inhibition and promotion dependent on the presence or absence of phase-variable pili. This unexpected mechanistic interdependence of local and invasive infection also suggests a new role of pilus phase variation in PorB_IA_-expressing gonococci as a stochastic event that controls the molecular switch to invasive gonococcal disease. Moreover, pilus expression in *N. gonorrhoeae* can irreversibly be lost [46], [47], a phenotype that – in the context of the PorB_IA_ allele – would favor the occurrence of invasive gonococcal infection.

## Materials and Methods

### Neisseria strains

*N. gonorrhoeae* MS11 derivatives used in this study are listed in table 2. N927 is a derivative of N138 with the *porB_IA_* gene of strain VP1 flanked by antibiotics resistance cassettes *cat* and *ermC* integrated into the *porB* locus. N927 in addition carries a deletion in the *pilE1* expression locus (non-revertible P^−^: Pn) [21]. N2009 (PorB_IA_, Opa^−^, P^+^) is a piliated variant of N138 expressing PorB_IA_ from N927. N2010 is a revertible non-piliated derivative of N2009 (PorB_IA_, Opa^−^,P^+^). N2013 was generated by transforming the PorB_IA_ expression cassette from strain N927 [21] into strain N503 (inducible *rec*A, *erm*C). Gonococci were routinely grown on GC agar base plates (Oxoid) supplemented with 1% vitamin mix for 14–18 h at 37°C in 5% CO_2_ in a humidified atmosphere. Opa and pili negative phenotypes were monitored by colony morphology under a stereo microscope or by immunoblotting.

**Table 2:** Neisseria MS11 derivatives.

Strains	Phenotype^a^	Genotype^b^, plasmid	Source
N927	PorB_IA_, Pn, P^−^, Opa^−^	ΔpilE1/2, cat<porBIA< >ermC	[21]
N138	PorB_IB_, P^+^, Opa^−^		[49]
N503	PorB_IB_, P^+^, Opa^−^	ΔpilE2, recA^I^, ermC	[50]
N2013	PorB_IA_, P^+^, Opa^−^	recA^I^	This study
N2015	PorB_IA_, P^−^, Opa^−^	recA^I^	This study
N2009	PorB_IA_, P^+^, Opa^−^	cat<porBIA< >ermC	This study
N2010	PorB_IA_, P^−^, Opa^−^	cat<porBIA< >ermC	This study
N924	PorB_IB_, Pn, P^−^, Opa^−^	pTH6	[21]
N313	PorB_IB_, Pn, P^−^, Opa_57_	pTH6 (opa57)	[27]
N931	PorB_IB_, Pn, P^−^, Opa_50_	pTH6 (opa50)	[51]

a Pn, non-revertible loss of piliation; P+, piliated; P−, non-piliated; Opa−, no detectable Opa expression;

b Shown are the genes and their orientation introduced into the genome of strain MS11; The plasmid pTH6 (with or without opa genes) are integrated into the Neisseria p*tetM*25.2 plasmid. Arrowheads indicate 5′ end (> or <) and 5′ to 3′ orientation (>) of genes.

### Infection conditions and gentamicin protection assay

2×10^5^ Chang cells were infected at an MOI 10, all other cells at an MOI 50 to achieve similar infection efficiency. Gentamicin protection assay was conducted as described [14]. Briefly, Chang cells were infected with the gonococcal strains at a confluency of 80–90%. To quantify total cell associated bacteria, cells were lysed with 1% saponin for 7 min. Suitable dilutions were plated on GC agar plates and CFU were determined 24 h later. For quantification of intracellular viable bacteria monolayers were incubated with 50 µg/ml gentamicin in HEPES medium for 2 h at 37°C and 5% CO_2_, prior to lysis in 1% saponin and plating. In general, 25–50% of the bacteria adhered to the cells (about 0.5–1×10^5^ gonococci per infection) and 10 and 20% of adherent PorB_IA_-expressing gonococci invaded the cells (1–2×10^4^ gonococci per infection).

### Immunoprecipitations

Active Rac1 was precipitated as described [48]. Briefly, cell lysates (5×10^6^ cells/each sample) were prepared in IP lysis buffer (50 mM Tris-HCl pH 7.5, 150 mM NaCl, 1% (v/v) Triton-X-100, 10% (v/v) glycerol, 2 mM EDTA, 25 mM NaF, and 2 mM NaH_2_PO_4_) containing protease and phosphatase inhibitor cocktails (Roche). Samples were incubated with 500 ng of anti-active- Rac1 monoclonal antibody (New East Bioscience) over night. Protein G Agarose (GE Healthcare) was added for 2 h. Caveolin was precipitated from lysates prepared in cell lysis buffer (20 mM Tris, 150 mM NaCl, 1 mM EDTA, 1 mM EGTA, 1% (v/v) Triton-X-100, 2.5 mM Sodiumpyrophosphate, 1 mM β-Glycerophosphate, pH 7.5) containing PhosStop Phosphatase Inhibitor and Complete Protease Inhibitor (Roche). The lysates were incubated with 2 µg bait antibody (anti-Caveolin, BD Transduction) overnight. Protein G-magnetic beads (Dynabeads, Invitrogen) were subsequently added for 4 h to precipitate antigen-antibody complexes. After extensive washing, the precipitate was eluted by heating to 95°C in SDS loading buffer and the individual proteins separated by SDS-PAGE. Western blotting was used to assess the precipitate.

### Western blotting

Cell lysates were resolved by 8–12% sodium dodecyl sulfate (SDS)-polyacrylamide gel electrophoresis. Proteins were transferred to polyvinylidene difluoride membranes (GE Healthcare) and blocked with Tris-buffered saline containing 0.1% Tween 20 and 3% bovine serum albumin. The following primary antibodies were used: anti-Flotillin, anti-PI3K (p85), anti-PI3K (p110), anti-pAKT, anti-Akt, anti-HA anti-PKD1, anti-pPKD1, anti-Vav2 (Cell Signaling), anti-Caveolin (BD Transduction), anti-SREC-I, anti-GST, anti-GFP, anti-PLCy1, anti-Tubulin (Santa Cruz Bioscience) and anti-Actin (Sigma Aldrich). Proteins were detected with peroxidase-coupled secondary antibodies using the ECL system (Pierce) and a Intas Chem HR 16-3200 reader and quantified by ImageJ software.

### Immunofluorescence staining

CHO cells or AGS cells were seeded onto cover slides, transfected with the indicated plasmids and 24 h after transfection infected under phosphate free conditions with N927 MOI 50 for 2 h. After extensive washing steps cells were fixed with 4% Paraformaldehyd for 15 min. For differentiating extra- from intracellular bacteria the staining method was used as described before [14]. Briefly, extracellular bacteria were detected with primary antibody, polyclonal rabbit anti-*N. gonorrhoeae* and subsequently samples were incubated with a Cy5 conjugated secondary anti-rabbit antibody. Cells were then permeabilized with 0.1% Triton-X-100 for 15 min. Staining of the extracellular and intracellular bacteria were subsequently performed as described above using a Cy3 conjugated secondary anti-rabbit antibody. Actin staining was carried out using Phalloidin-647 (MFP) 100 nM. Bacteria were stained before infection with the fluorescent dye SNARF (Invitrogen) for 20 min and washed several times with infection medium before infection.

## Supporting Information

Dataset S1 **Supporting data set.** This data set shows the result of a statistical analysis on the basis of published data to demonstrate the association of PorB_IA_-expressing strains with DGI.
(RTF)

Figure S1 **Surface expression of SREC-I and truncated SREC-I on CHO cells.** Surface exposed SREC-I on CHO cells transfected with empty vector control (pEGFP-N1), SREC-I wt or truncated SREC-I constructs was detected via FACS analysis by incubation with monoclonal anti-SREC-I antibody and an anti-mouse Cy5-conjugated antibody. The APC-A axis indicates surface exposed SREC-I fluorescence. Data are representative for three independent experiments.
(TIF)

Figure S2 **Interaction of SREC-I with N927 requires intact membrane rafts.** (**A**) Chang cells were either left untreated (-) or were pretreated for 1 h with 50 µg/ml Nystatin and then infected with N927 at an MOI of 10 for 30 min. Intracellular (white bars inv) and adherent (black bars adh) bacteria were quantified by gentamicin protection assays and the number bacteria recovered from untreated control cells was set to 100%. Shown is the mean ± SD of three independent experiments each performed in duplicate. p<0.01: ** (**B**) Chang cells were pretreated for 1 h with 25 µg/ml or 50 µg/ml Nystatin and then infected with N927 at an MOI of 10 for 30 min. Analysis was performed as described in (B). (**C**) Reduced recruitment of SREC-I by N927 after membrane raft disruption. Chang cells were treated with 50 µg/ml Nystatin for 1 h before infection with N927 (PorB_IA_, P^−^) at MOI 25. Bacteria were visualized by SNARF-1 staining and SREC-I was detected with a polyclonal serum against SREC-I (Imagenex) and a Cy2-conjugated secondary antibody. Co-localization of SREC-I and gonococci (white arrows) was analyzed by confocal immunofluorescence microscopy. Scale bar: 10 µm. (**D**) Chang cells were treated or not with 5 mg/ml Methyl-β-cyclodextrin (MβCD). Cells were either infected for 30 min immediately after the 30 min MβCD treatment or 3 h after replacement of MβCD by regular growth medium (wash out). The number of adherent and intracellular bacteria was determined by gentamicin protection assay and the number of adherent and invasive bacteria of the untreated control was set to 100%. Experiments were performed four times each in duplicates. p<0.01: **, p<0.05: *.
(TIF)

Figure S3 **PorB_IA_-triggered invasion depends on Cav1 pY14 and the switch-off of pilus production.** (**A**) Depiction of figure 2D from the main manuscript presented with separate channels. AGS Cav1 or AGS Cav1Y14F were infected with N927 at MOI 25. Adherent (blue and red) and intracellular (red) bacteria were detected by differential immunofluorescence assay. Caveolin expression was visualized with an HA antibody and a Cy2-conjugated secondary antibody (green). (**B**) Cytoskeletal rearrangements after infection: Chang cells were infected with either N927 (PorB_IA_, P^−^) or N138 (PorB_IB_, P^+^) at an MOI of 25 for 30 min under phosphate free conditions. Cells were fixed and actin was stained with Phalloidin 647 (MFP, green). (**C**) Only piliated gonococci form microcolonies. Cells were infected as under (B) and gonococci (red) were additionally stained with anti-Ngo rabbit IgG (US Biological) and secondary Cy3 anti-rabbit antibody. (**D**) N138 fails to invade Chang cells. Chang cells were infected at an MOI of 10 for 30 min with either N927 or N138. Adherence (white bars) and invasion (black bars) was analyzed by gentamicin protection assays. The number of adherent and invasive bacteria of the strain N927 was set to 100%. Experiments were performed three times each in duplicates. p<0.01: **.
(TIF)

Figure S4 **PLCγ1 but not Vav2 is essential for N927 invasion.** (**A**) Validation of PLCγ1 silencing. shRNA-mediated downregulation of PLCγ1 in HeLa cells was quantified by Western blot. (**B**) shRNA-mediated downregulation of Vav2 in Hela cells has no effect on internalization of N927 (PorB_IA_, P^−^). Control cells (shLuci) as well as shVav2 cells (shVav2) were infected with strain N927 (MOI 10; 30 min) and adherence (white bars) as well as invasion (black bars) were analyzed by gentamicin protection assay. The number of adherent and invasive bacteria of control cells (shLuci) was set to 100%. Shown are mean values ± SD of three independent experiments done in duplicates. (**C**) Knock down of Vav2 in Hela cells was verified by Western blotting. Actin was detected as loading control.
(TIF)

Figure S5 **Infection-induced activation of PI3K.** (**A**) Cav1-deficient AGS cells as well as Cav1-expressing transgenic AGS cells were either infected with N927 (PorB_IA_, P^−^) or N138 (PorB_IB_, P^+^) at an MOI of 50 for 30 min. PI3K activity was analyzed by immunoblotting using anti-pAKT antibody. (**B**) Relative amount of pAKT quantified from the experiment shown in (A). (**C**) Chang cells were infected with strains N931 (PorB_IB_, P^−^, Opa_50_), N313 (PorB_IB_, P^−^, Opa_57_), N924 (PorB_IB_, P^−^, Opa^−^) and N927 (PorB_IA_, P^−^, Opa^−^) at an MOI of 75 for 30 min and PI3K activity was determined by immunoblotting using anti-pAKT antibody. (**D**) The strains used for infection in (C) were tested for the PorB subtype. Gonococci were lysed, separated by SDS-Page and analyzed by Coomassie staining. As PorB is the major outer membrane protein a prominent band is visible at 35 kDa (PorB_IB_ subtype) or at 34 kDa (PorB_IA_ subtype). (**E**) End1 cells were pretreated for 1 h with PI3K inhibitor LY294002 (LY, 10 µM)) and infected with N927 (MOI 50) for 30 min. Adherence (white bars) and invasion (black bars) were quantified by gentamicin protection assay. The number of adherent and invasive bacteria of untreated control cells was set to 100%. The graph shows mean values ± SD of three independent experiments performed in duplicates. p<0.01: ** (**F**) Activation of PI3K shown by phosphorylation of Akt. Whole cell lysates of End1 cells infected with either N927 or N138 at MOI 50 were subjected to SDS PAGE and Western blotting using anti-phospho-Akt, anti-Akt and anti-Actin antibodies.
(TIF)

Figure S6 **PKD1 is involved in PorB_IA_- and Opa_50_-mediated invasion.** (**A,B**) Chang cells were transfected with siRNAs against PKD1/PKCμ and luciferase as control. The cells were infected with N927 (PorB_IA_, P^−^, Opa^−^ MOI 10), N931 (PorB_IB_, P^−^, Opa_50_ MOI 50) and N313 (PorB_IB_, P^−^, Opa_57_ MOI 50) for 120 min under low phosphate conditions 72 h after siRNA transfection. Adherent (A) and intracellular (B) bacteria were quantified by gentamicin protection assay. Shown are the means ± SD of three independent experiments done in duplicates. Invasion in and adherence to control cells transfected with siLuc was set to 100%. p<0.01: **.
(TIF)

Figure S7 **Cytotoxic effect of chemical inhibitors tested under assay conditions.** Cytotoxicity of inhibitors was tested in Chang cells by propidium iodide uptake assay. Chang cells were treated with the different inhibitors at the indicated concentrations (Nystatin 50 µg/ml, U73122 10 µM, LY294002 10 µM, Imatinib 10 µM, NSC23766 100 µM, Gö6976 3 µM, MβCD 5 mg/ml) for 1 h. Triton (0.1%) was used as positive control and was added 5 min before analysis. Flow cytometry analysis was performed after staining with PI. The FL2-A axis indicates PI fluorescence.
(TIF)

Figure S8 **SREC-I surface expression after inhibitor treatment.** SREC-I was detected on Chang cells by incubation with an anti-SREC-I antibody or an isotype control after treatment with the respective inhibitors (**A**) dissolved in water, (**B**) dissolved in DMSO or (**C**) in Hepes medium. As secondary antibody Cy2-labeled anti-mouse antibody was used. Graphs show the mean fluorescence and represent means ± SD of three independent experiments. P-values refer to untreated control cells.
(TIF)

Figure S9 **Piliation affects invasion.** (**A**) Piliation status of N2009 was verified by electron micrographs. Photographs were taken at 12,500- or 20,000-fold magnifications. (**B**) N2009 (PorB_IA_, P^+^) failed to efficiently invade End1 cells. End1 cells were infected at an MOI of 10 for 30 min with either N2010 (PorB_IA_, P^−^) or N2009. Adherent (white bars) and intracellular bacteria (black bars) were counted from 50 randomly chosen cells using differential immunostaining and confocal microscopy. Shown is the mean ± SD of three independent experiments. The number of adherent and invasive bacteria of strain N2010 was set to 100%. p<0.01: **.
(TIF)

Text S1 **Supplementary materials and methods.** Additional information on cell culture and transfection, infection at low phosphate concentrations, DNA constructs, peptide synthesis and streptavidin-agarose pull-down, generation of stable PLCγ1 knockdown cell lines, detection of surface exposed SREC-I on CHO cells by FACS-analysis, electron microscopy, purification of caveolin-rich membrane fractions and a Cytotoxicity assay. Supplementary Table S1: Primary antibodies for immunoblotting (IB), immunofluorescence (IF), immunoprecipitation (IP) or flow cytometry (FACS) are listed.
(RTF)
