# Supplementary material for: Pilus Phase Variation Switches Gonococcal Adherence to Invasion by Caveolin-1-Dependent Host Cell Signaling
Source: PLoS Pathog. 2013 May 23;9(5):e1003373. doi: 10.1371/journal.ppat.1003373 (PMC3662692; doi:10.1371/journal.ppat.1003373)
Supplement: Dataset S1 — Supporting data set. This data set shows the result of a statistical analysis on the basis of published data to demonstrate the association of PorBIA-expressing strains with DGI. (RTF) [file ppat.1003373.s001.rtf]

Dataset S1:
Gonococci express only one PorB porin of either type A or B. Deletion mutants in PorB are not viable implying that porB genes identified by sequencing are expressed. The following available datasets were used to test the correlation of disseminated gonococcal infections (DGI) with frequencies of porB alleles: 
DGI-strains
	Number of isolates	PorBIA	PorBIB	Time period	publication	
Seattle, Denver and Atlanta	101	85	26	1971-1973 and 1980-1982	(Sandstrom et al., 1984)	
Boston City and University Hospitals 	53	42	11	1975-1982	(Bash et al., 2005)	
Lübeck and Heidelberg, Germany	7	6	1	1976-1978 and 1980-1982	(Kohl et al., 1985)	
University of Chicago Medical Center	86	77	9	1973-1983	(Morello and Bohnhoff, 1989)	
Darwin, Queensland, Papua New Guinea, Sydney	22	21	1	1985-1991	(Tapsall et al., 1992)	

Non-DGI-strains
	Number of isolates	PorBIA	PorBIB	Time period	publication	
Seattle, Denver and Atlanta	168	67	101	1971-1973 and 1980-1982	(Sandstrom et al., 1984)	
Boston City and University Hospitals 	109	28	81	1975-1982	(Bash et al., 2005)	
Lübeck and Heidelberg, Germany	49	20	29	1976-1978 and 1980-1982	(Kohl et al., 1985)	
University of Chicago Medical Center	137	36	101	1973-1983	(Morello and Bohnhoff, 1989)	
Darwin, Queensland, Papua New Guinea, Sydney	35	19	16	1985-1991	(Tapsall et al., 1992)	

We then tested the significance of association of either porB allele with DGI and non-DGI disease outcomes, respectively. For that purpose we employed the Fisher Test using the software SPSS. In single studies as well as in the combination of all studies there was a significant correlation of PorBIA with the DGI disease. 

	Seattle, Denver,
Atlanta	Boston City and University Hospitals	Lübeck and Heidelberg, Germany	University of Chicago Medical Center	Darwin, Queensland, Papua New Guinea, Sydney	total	
p-values	p<0,001	p<0,001	p<0,05	p<0,001	p<0,001	p<0,001	

Literature
Bash, M.C., Zhu, P., Gulati, S., McKnew, D., Rice, P.A., and Lynn, F. (2005). por Variable-region typing by DNA probe hybridization is broadly applicable to epidemiologic studies of Neisseria gonorrhoeae. J Clin Microbiol 43, 1522-1530.
Kohl, P.K., Meyer, T.F., and Petzoldt, D. (1985). [Gonococcal surface antigens and their significance for serotyping and vaccines]. Hautarzt 36, 320-325.
Morello, J.A., and Bohnhoff, M. (1989). Serovars and serum resistance of Neisseria gonorrhoeae from disseminated and uncomplicated infections. J Infect Dis 160, 1012-1017.
Sandstrom, E.G., Knapp, J.S., Reller, L.B., Thompson, S.E., Hook, E.W., 3rd, and Holmes, K.K. (1984). Serogrouping of Neisseria gonorrhoeae: correlation of serogroup with disseminated gonococcal infection. Sex Transm Dis 11, 77-80.
Tapsall, J.W., Phillips, E.A., Shultz, T.R., Way, B., and Withnall, K. (1992). Strain characteristics and antibiotic susceptibility of isolates of Neisseria gonorrhoeae causing disseminated gonococcal infection in Australia. Members of the Australian Gonococcal Surveillance Programme. Int J STD AIDS 3, 273-277.
