# Supplementary figures and images for: Pilus Phase Variation Switches Gonococcal Adherence to Invasion by Caveolin-1-Dependent Host Cell Signaling
Source: PLoS Pathog. 2013 May 23;9(5):e1003373. doi: 10.1371/journal.ppat.1003373 (PMC3662692; doi:10.1371/journal.ppat.1003373)

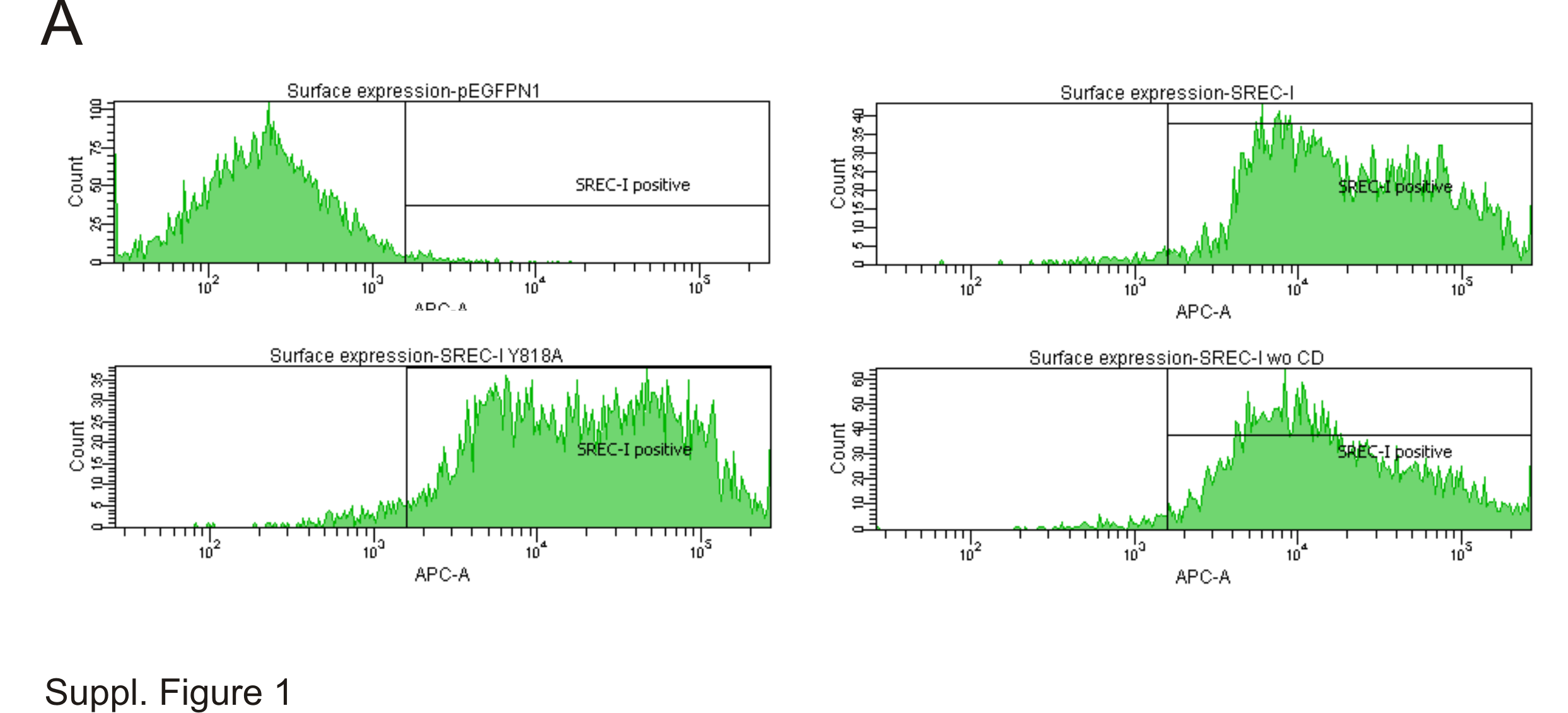

Supplement: Figure S1 — Surface expression of SREC-I and truncated SREC-I on CHO cells. Surface exposed SREC-I on CHO cells transfected with empty vector control (pEGFP-N1), SREC-I wt or truncated SREC-I constructs was detected via FACS analysis by incubation with monoclonal anti-SREC-I antibody and an anti-mouse Cy5-conjugated antibody. The APC-A axis indicates surface exposed SREC-I fluorescence. Data are representative for three independent experiments. (TIF) [file ppat.1003373.s002.tif]

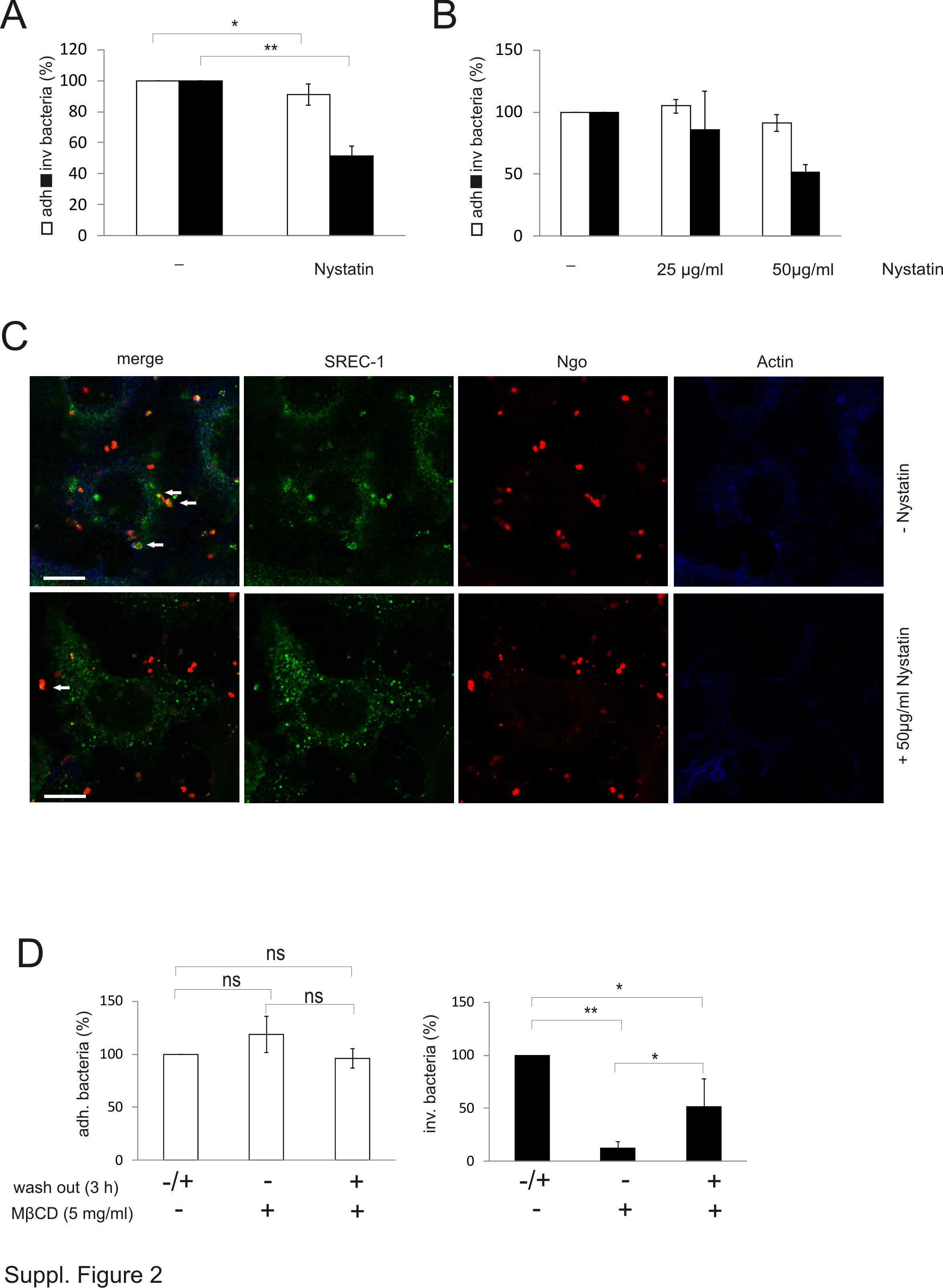

Supplement: Figure S2 — Interaction of SREC-I with N927 requires intact membrane rafts. (A) Chang cells were either left untreated (-) or were pretreated for 1 h with 50 µg/ml Nystatin and then infected with N927 at an MOI of 10 for 30 min. Intracellular (white bars inv) and adherent (black bars adh) bacteria were quantified by gentamicin protection assays and the number bacteria recovered from untreated control cells was set to 100%. Shown is the mean ± SD of three independent experiments each performed in duplicate. p<0.01: ** (B) Chang cells were pretreated for 1 h with 25 µg/ml or 50 µg/ml Nystatin and then infected with N927 at an MOI of 10 for 30 min. Analysis was performed as described in (B). (C) Reduced recruitment of SREC-I by N927 after membrane raft disruption. Chang cells were treated with 50 µg/ml Nystatin for 1 h before infection with N927 (PorBIA, P−) at MOI 25. Bacteria were visualized by SNARF-1 staining and SREC-I was detected with a polyclonal serum against SREC-I (Imagenex) and a Cy2-conjugated secondary antibody. Co-localization of SREC-I and gonococci (white arrows) was analyzed by confocal immunofluorescence microscopy. Scale bar: 10 µm. (D) Chang cells were treated or not with 5 mg/ml Methyl-β-cyclodextrin (MβCD). Cells were either infected for 30 min immediately after the 30 min MβCD treatment or 3 h after replacement of MβCD by regular growth medium (wash out). The number of adherent and intracellular bacteria was determined by gentamicin protection assay and the number of adherent and invasive bacteria of the untreated control was set to 100%. Experiments were performed four times each in duplicates. p<0.01: **, p<0.05: *. (TIF) [file ppat.1003373.s003.tif]

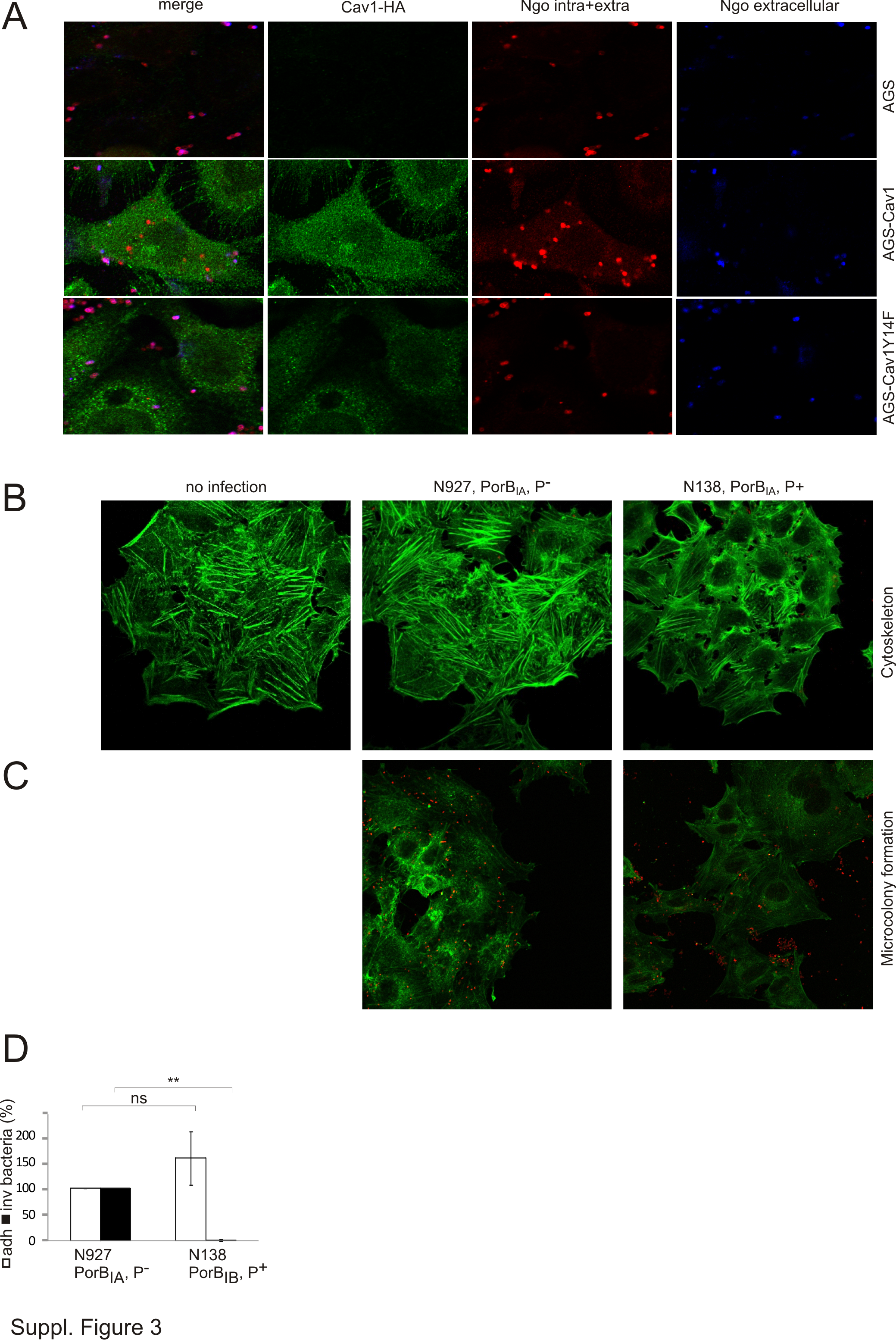

Supplement: Figure S3 — PorBIA-triggered invasion depends on Cav1 pY14 and the switch-off of pilus production. (A) Depiction of figure 2D from the main manuscript presented with separate channels. AGS Cav1 or AGS Cav1Y14F were infected with N927 at MOI 25. Adherent (blue and red) and intracellular (red) bacteria were detected by differential immunofluorescence assay. Caveolin expression was visualized with an HA antibody and a Cy2-conjugated secondary antibody (green). (B) Cytoskeletal rearrangements after infection: Chang cells were infected with either N927 (PorBIA, P−) or N138 (PorBIB, P+) at an MOI of 25 for 30 min under phosphate free conditions. Cells were fixed and actin was stained with Phalloidin 647 (MFP, green). (C) Only piliated gonococci form microcolonies. Cells were infected as under (B) and gonococci (red) were additionally stained with anti-Ngo rabbit IgG (US Biological) and secondary Cy3 anti-rabbit antibody. (D) N138 fails to invade Chang cells. Chang cells were infected at an MOI of 10 for 30 min with either N927 or N138. Adherence (white bars) and invasion (black bars) was analyzed by gentamicin protection assays. The number of adherent and invasive bacteria of the strain N927 was set to 100%. Experiments were performed three times each in duplicates. p<0.01: **. (TIF) [file ppat.1003373.s004.tif]

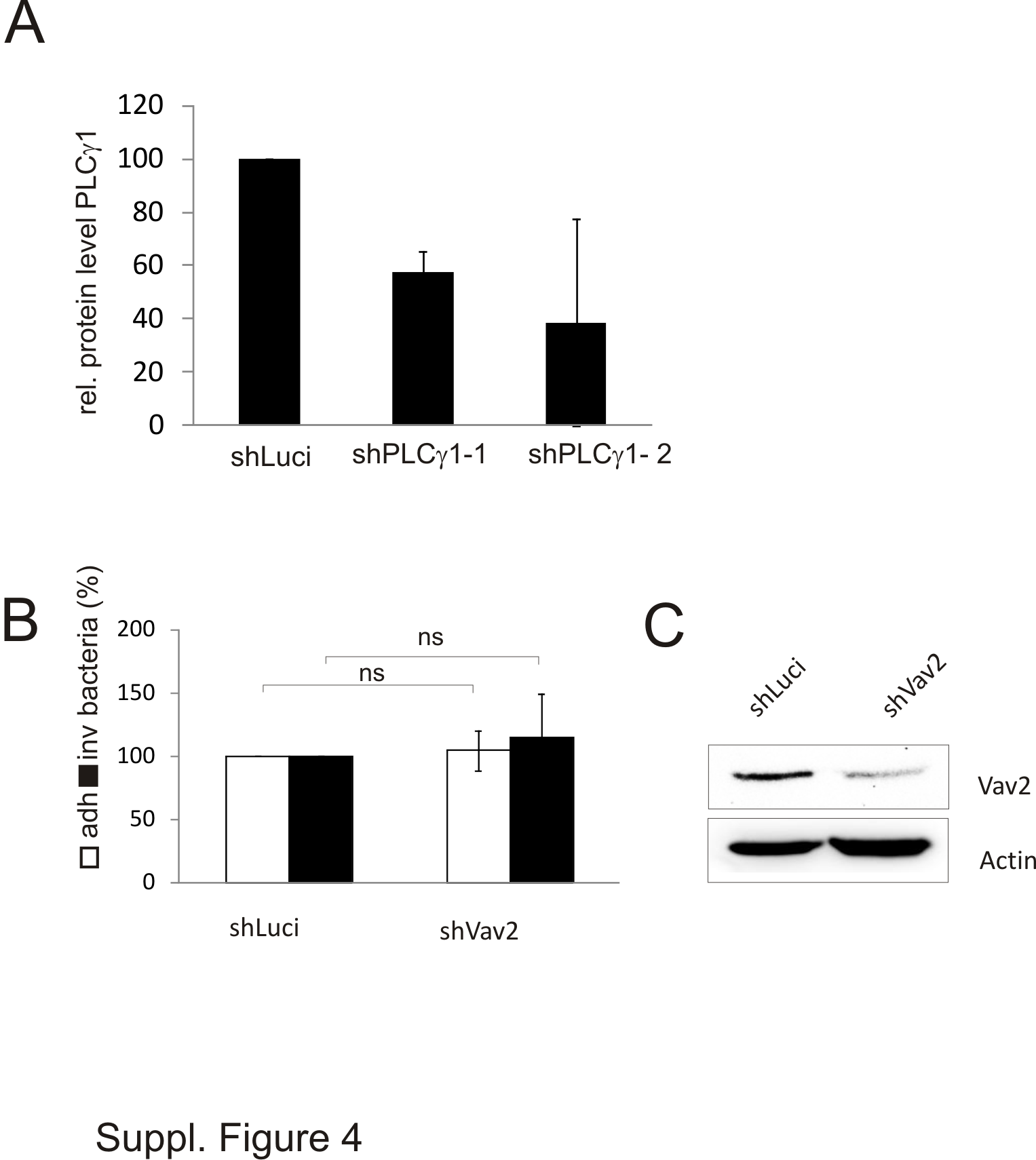

Supplement: Figure S4 — PLCγ1 but not Vav2 is essential for N927 invasion. (A) Validation of PLCγ1 silencing. shRNA-mediated downregulation of PLCγ1 in HeLa cells was quantified by Western blot. (B) shRNA-mediated downregulation of Vav2 in Hela cells has no effect on internalization of N927 (PorBIA, P−). Control cells (shLuci) as well as shVav2 cells (shVav2) were infected with strain N927 (MOI 10; 30 min) and adherence (white bars) as well as invasion (black bars) were analyzed by gentamicin protection assay. The number of adherent and invasive bacteria of control cells (shLuci) was set to 100%. Shown are mean values ± SD of three independent experiments done in duplicates. (C) Knock down of Vav2 in Hela cells was verified by Western blotting. Actin was detected as loading control. (TIF) [file ppat.1003373.s005.tif]

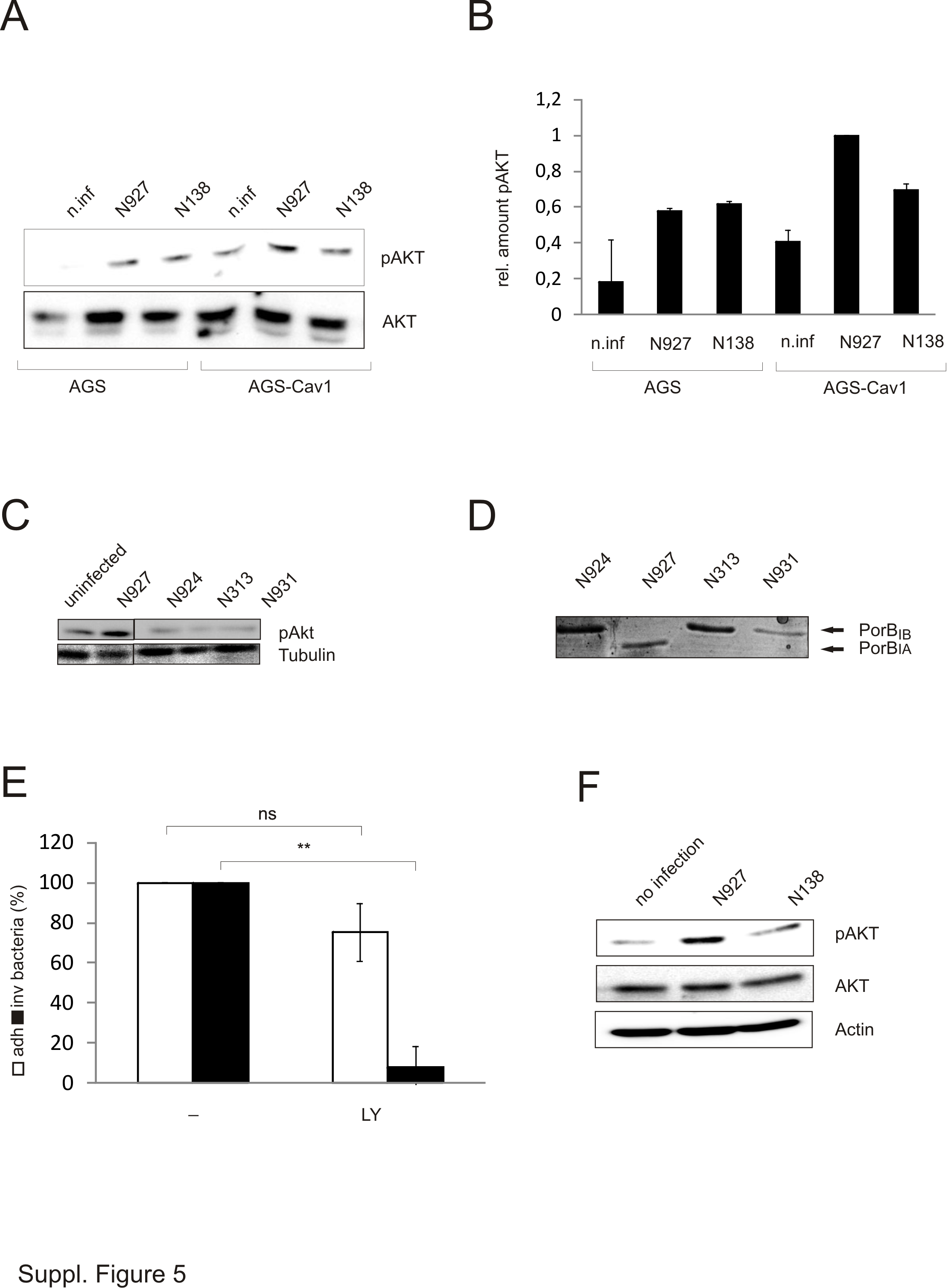

Supplement: Figure S5 — Infection-induced activation of PI3K. (A) Cav1-deficient AGS cells as well as Cav1-expressing transgenic AGS cells were either infected with N927 (PorBIA, P−) or N138 (PorBIB, P+) at an MOI of 50 for 30 min. PI3K activity was analyzed by immunoblotting using anti-pAKT antibody. (B) Relative amount of pAKT quantified from the experiment shown in (A). (C) Chang cells were infected with strains N931 (PorBIB, P−, Opa50), N313 (PorBIB, P−, Opa57), N924 (PorBIB, P−, Opa−) and N927 (PorBIA, P−, Opa−) at an MOI of 75 for 30 min and PI3K activity was determined by immunoblotting using anti-pAKT antibody. (D) The strains used for infection in (C) were tested for the PorB subtype. Gonococci were lysed, separated by SDS-Page and analyzed by Coomassie staining. As PorB is the major outer membrane protein a prominent band is visible at 35 kDa (PorBIB subtype) or at 34 kDa (PorBIA subtype). (E) End1 cells were pretreated for 1 h with PI3K inhibitor LY294002 (LY, 10 µM)) and infected with N927 (MOI 50) for 30 min. Adherence (white bars) and invasion (black bars) were quantified by gentamicin protection assay. The number of adherent and invasive bacteria of untreated control cells was set to 100%. The graph shows mean values ± SD of three independent experiments performed in duplicates. p<0.01: ** (F) Activation of PI3K shown by phosphorylation of Akt. Whole cell lysates of End1 cells infected with either N927 or N138 at MOI 50 were subjected to SDS PAGE and Western blotting using anti-phospho-Akt, anti-Akt and anti-Actin antibodies. (TIF) [file ppat.1003373.s006.tif]

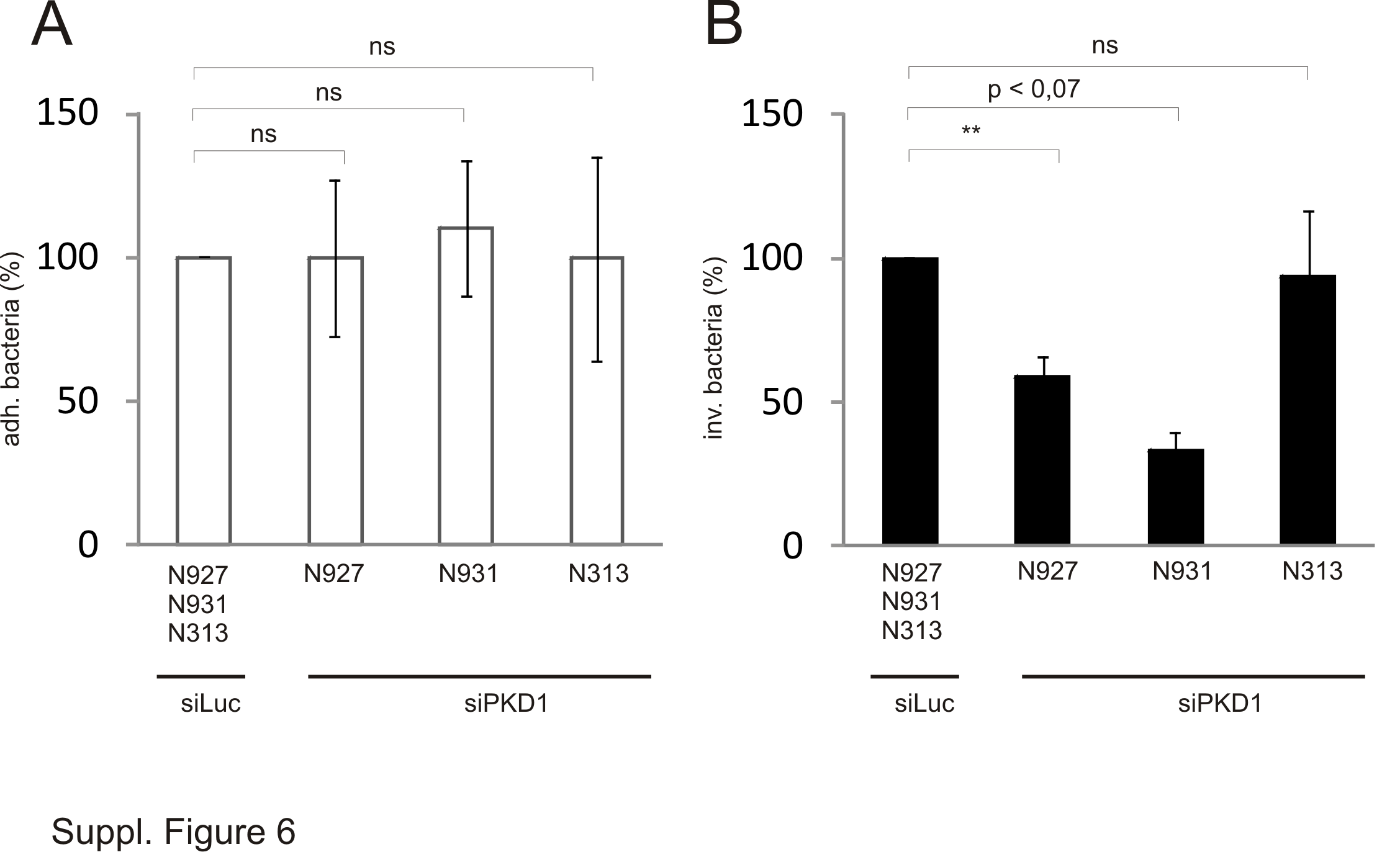

Supplement: Figure S6 — PKD1 is involved in PorBIA- and Opa50-mediated invasion. (A,B) Chang cells were transfected with siRNAs against PKD1/PKCμ and luciferase as control. The cells were infected with N927 (PorBIA, P−, Opa− MOI 10), N931 (PorBIB, P−, Opa50 MOI 50) and N313 (PorBIB, P−, Opa57 MOI 50) for 120 min under low phosphate conditions 72 h after siRNA transfection. Adherent (A) and intracellular (B) bacteria were quantified by gentamicin protection assay. Shown are the means ± SD of three independent experiments done in duplicates. Invasion in and adherence to control cells transfected with siLuc was set to 100%. p<0.01: **. (TIF) [file ppat.1003373.s007.tif]

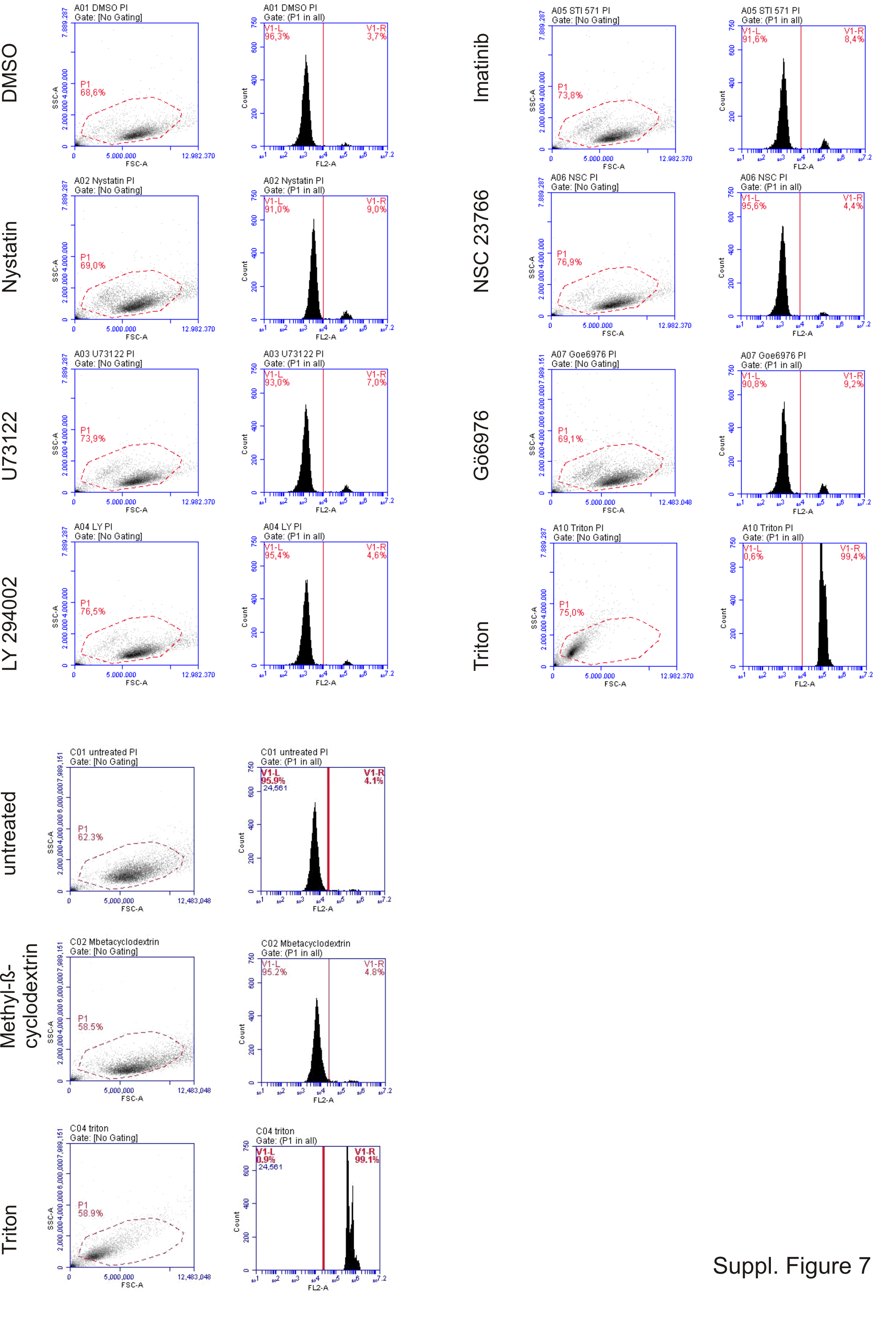

Supplement: Figure S7 — Cytotoxic effect of chemical inhibitors tested under assay conditions. Cytotoxicity of inhibitors was tested in Chang cells by propidium iodide uptake assay. Chang cells were treated with the different inhibitors at the indicated concentrations (Nystatin 50 µg/ml, U73122 10 µM, LY294002 10 µM, Imatinib 10 µM, NSC23766 100 µM, Gö6976 3 µM, MβCD 5 mg/ml) for 1 h. Triton (0.1%) was used as positive control and was added 5 min before analysis. Flow cytometry analysis was performed after staining with PI. The FL2-A axis indicates PI fluorescence. (TIF) [file ppat.1003373.s008.tif]

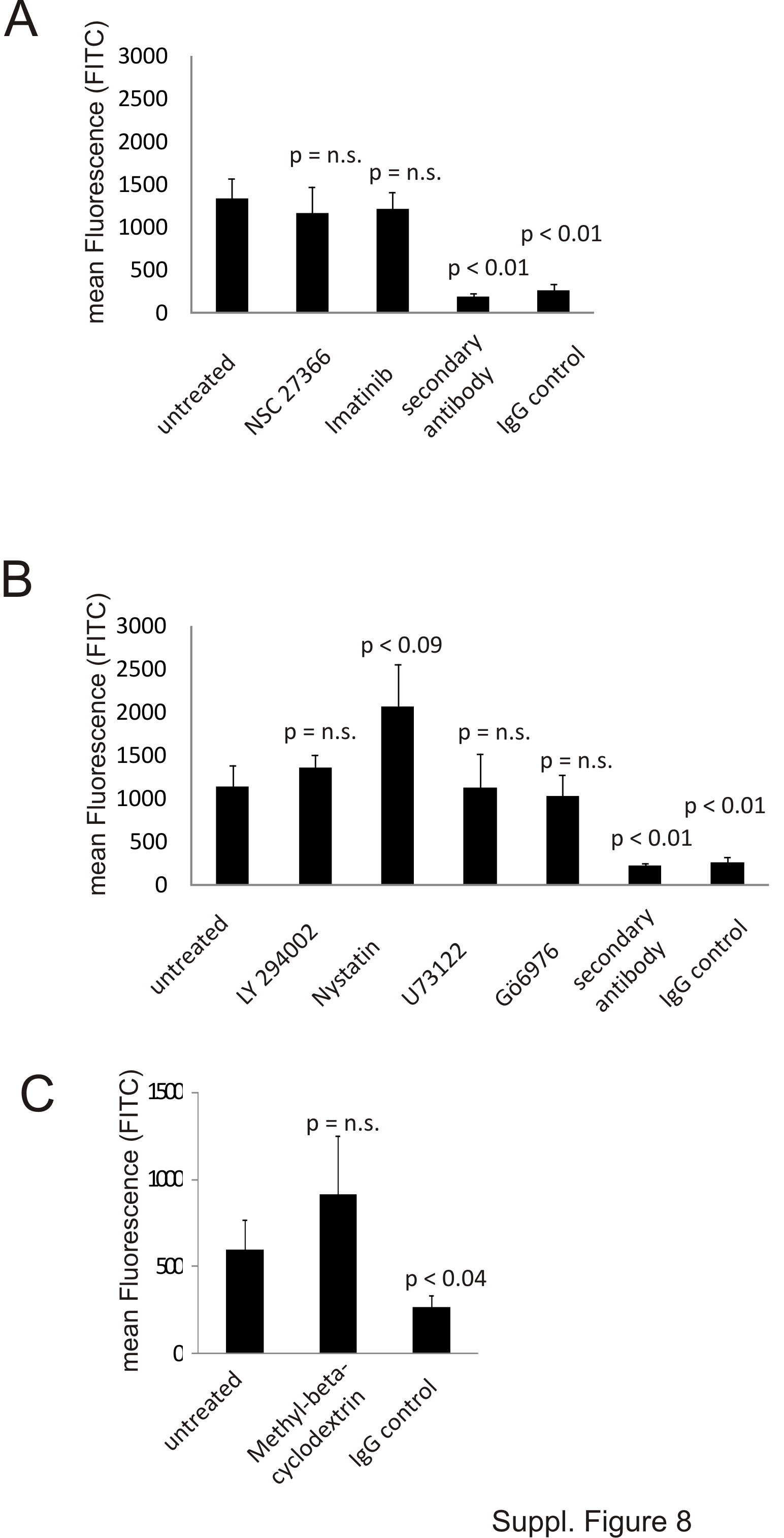

Supplement: Figure S8 — SREC-I surface expression after inhibitor treatment. SREC-I was detected on Chang cells by incubation with an anti-SREC-I antibody or an isotype control after treatment with the respective inhibitors (A) dissolved in water, (B) dissolved in DMSO or (C) in Hepes medium. As secondary antibody Cy2-labeled anti-mouse antibody was used. Graphs show the mean fluorescence and represent means ± SD of three independent experiments. P-values refer to untreated control cells. (TIF) [file ppat.1003373.s009.tif]

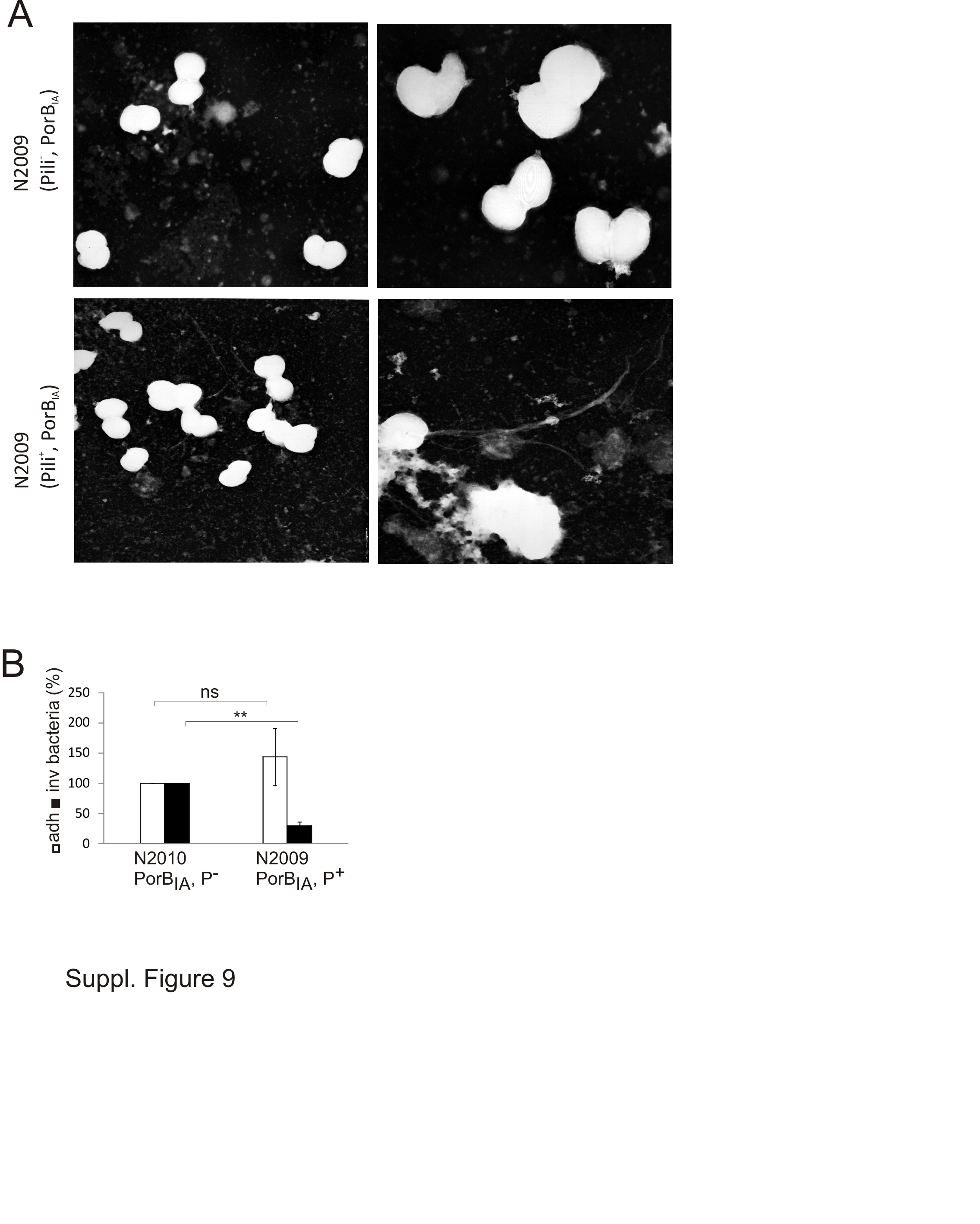

Supplement: Figure S9 — Piliation affects invasion. (A) Piliation status of N2009 was verified by electron micrographs. Photographs were taken at 12,500- or 20,000-fold magnifications. (B) N2009 (PorBIA, P+) failed to efficiently invade End1 cells. End1 cells were infected at an MOI of 10 for 30 min with either N2010 (PorBIA, P−) or N2009. Adherent (white bars) and intracellular bacteria (black bars) were counted from 50 randomly chosen cells using differential immunostaining and confocal microscopy. Shown is the mean ± SD of three independent experiments. The number of adherent and invasive bacteria of strain N2010 was set to 100%. p<0.01: **. (TIF) [file ppat.1003373.s010.tif]
