# Supplementary material for: Pilus Phase Variation Switches Gonococcal Adherence to Invasion by Caveolin-1-Dependent Host Cell Signaling
Source: PLoS Pathog. 2013 May 23;9(5):e1003373. doi: 10.1371/journal.ppat.1003373 (PMC3662692; doi:10.1371/journal.ppat.1003373)
Supplement: Text S1 — Supplementary materials and methods. Additional information on cell culture and transfection, infection at low phosphate concentrations, DNA constructs, peptide synthesis and streptavidin-agarose pull-down, generation of stable PLCγ1 knockdown cell lines, detection of surface exposed SREC-I on CHO cells by FACS-analysis, electron microscopy, purification of caveolin-rich membrane fractions and a Cytotoxicity assay. Supplementary Table S1: Primary antibodies for immunoblotting (IB), immunofluorescence (IF), immunoprecipitation (IP) or flow cytometry (FACS) are listed. (RTF) [file ppat.1003373.s011.rtf]

Text S1
Supplementary Materials and Methods
Cell culture and transfection 
Human Chang human conjunctiva cells (ATCC CCL20.2) were cultured in RPMI (Gibco), CHO-K1 Chinese hamster ovary cells (ATCC CCL61) in Ham´s F12 medium (Gibco) and AGS cells in DMEM (Gibco). End1 cells (ATCC CRL-2614) were cultured in a medium containing 1:1 mixture of Dulbecco's modified Eagle's medium and Ham's F12 medium. Media were supplemented with 10% fetal calf serum and cells were grown at 37C in 5% CO2. The generation of stable AGS-Cav1 clones has been described [1]. CHO-K1, Chang and AGS cells were transfected with Lipofectamine (Invitrogen) according to the manufacturers' instructions. Chang cells were transfected with siRNA at a concentration of 25 nM. Transfected cells were subcultured after 24 h and analyzed after 72 h. Stable CHO-K1 cells were generated by transfection with vector pEGFP-N1 or pEGFP-N1-SREC-I construct, then stable single cell clones were isolated and maintained in F12 Ham's Medium supplemented with 10% FCS and 400 µg/ml G418.

Infection at low phosphate concentrations
Cells grown to 80% confluency were routinely infected in phosphate-free HEPES medium as previously described [2]. The monolayers were washed once with HEPES medium to remove phosphate before infection. Bacteria were suspended in HEPES medium and infection was started by centrifugation for 3 min at 600 × g. Infected cells were cultured at 37°C in 5% CO2 for different time periods (Chang cells 30 min; AGS cells 1 h; CHO cells 2 h) and infection was stopped by washing the cells several times with HEPES medium. For inhibitor studies, cells were incubated with the inhibitors 30 - 60 min prior to infection in HEPES medium. A toxic effect on gonococci in HEPES medium was excluded for all inhibitors. In addition, cytotoxic effects (Fig. S7) or downregulation of SREC-I (Fig. S8) were ruled out for all inhibitors. 

DNA constructs
The GFP-tagged version of SREC-I was constructed by amplifying the cDNA-clone of wt-SREC-I (IRATp970A0758D6, RZPD: Deutsches Ressourcenzentrum für Genforschung GmbH) with primers SREC-I sense 5'-GACTAGATCTGACGAACCCGAGCGCTGCCACCG-3' and SREC-I antisense 5'-GACTGAATTCGTTCTGTTGGCCTGGAGATGG-3'. The resulting PCR fragments were cloned into pGEM-Teasy TA-vector system (Promega) and transferred by BglII /EcoRI digestion and ligation into pEGFP-N1 (Clontech) resulting in GFP fused to the carboxy-terminus of the expressed proteins. SREC-IY818A was constructed by site-directed mutagenesis using SREC-I as a template and the specific mutagenesis primer pairs sense 5'- GAGGAACCTGAGGCTGAGAATGTT -3' and antisense 5'- AACATTCTCAGCCTCAGGTTCCTC -3'. SREC-IÄCD was generated from SREC-I wt template using the DiTriSEC method (Steffen, 1996 Roche PCR Applications Manual, Roche Molecular Biochemicals) and pEGFP-N1 as a vector. The respective primers were sense 5'-ATCGATGGGGCTGGGGCTGCTGCT-3' and antisense 5'-AATTGAGAGGTCTGATCGGGGGGC-3'. 
For overexpression of PKD1, a HA-tagged construct was used (Addgene Plasmid 10808: [3]). Caveolin-HA and Cav1Y14F were described before [1].  

Peptide Synthesis and Streptavidin-Agarose Pull-Down 
Peptides were designed as 15-mers (residues 7–21 of Cav1) bearing an N-terminal biotin. Peptides were synthesized as pairs, one phosphorylated on Tyr14 and the other not phosphorylated, purified to > 95% by preparative reverse phase HPLC, and quality controlled via mass spectrometry and analytical HPLC. For affinity pull-downs, 10 nmol of immobilized peptide was added to ~2 mg of cell lysate. Cells were lysed in 1× immunoprecipitation cell lysis buffer containing 2 mM sodium orthovanadate, as a phosphatase inhibitor, and Complete Protease Inhibitor. The lysates were pre-cleared for 1 h with streptavidin agarose beads and equal amounts of lysate were incubated overnight at 4 °C with streptavidin agarose beads, pre-saturated with the respective biotinylated peptides. After extensive washing, the streptavidin precipitate was eluted by heating to 95 °C in SDS loading buffer and the individual proteins separated by SDS-PAGE. Separated protein bands of interest were identified by MALDI-TOF-TOF using a 4700 Proteomics Analyzer. Additionally, Western blotting was used to assess the precipitate.

Generation of stable PLCã1 knockdown cell lines
Stable, shRNA-mediated knockdown of human phospholipase Cã1 (PLCã1) (transcript variant 1, NM_002660) as well as Vav2 was achieved with a lentiviral expression system [4]. In brief, oligonucleotides were designed using BLOCK-iT™ RNAi Designer (Invitrogen, USA) and synthesized by Metabion, Germany with the following sequences: 5'-GCCATACTTCTTCCTGGATGA-3' (PLCã1-1), 5'-GCCTGTTCCCTTTGATGAAGA-3' (PLCã1-2) and 5'-GCATGACTGAAGATGACAAGA-3' (Vav-2). Oligonucleotides were cloned into the lentiviral vector pLVTHM and constructs were validated by sequencing. Viruses carrying the shRNAs were produced by transfecting 293T cells with the pLVTHM constructs in combination with viral packaging vectors (psPAX2, pMD2G) by calcium phosphate transfection. 48 h after transfection, viruses were harvested from the supernatant, filtrated through a 0.45 µm filter and applied to HeLa229 cells in the presence of polybrene (10 µg/ml, Sigma-Aldrich, USA) for lentiviral infection. Cells expressing shRNA against a firefly luciferase (target sequence AACUUACGCUGAGUACUUCGA) were used as control. Efficiency of gene silencing was verified by Western blotting. Vectors were kindly provided by Didier Trono (Ecole Polytechnique Fédérale de Lausanne, Switzerland).

Detection of surface exposed SREC-I by FACS analysis
CHO cells were transfected with vector pEGFP-N1 or pEGFP-N1-SREC-I constructs. 24 h post transfection cells (5x10^5^) were detached by scraping in PBS supplemented with 1% FCS. Cells were blocked with 10% FCS in PBS for 20 min at room temperature following incubation with the anti-SREC-I antibody (R&D) for 45 min at 4°C. Then, cells were washed twice and incubated for 45 min with secondary antibody (anti-mouse Cy5). 
Chang cells cultured to 80% confluence were treated with the indicated concentrations of inhibitors or DMSO as a control for 1 h. Cells were detached with trypsin, fixed with 4% paraformaldeyde in PBS for 15 min at room temperature. PFA was removed and cells were washed once with 10% FCS in PBS following incubation with the anti-SREC-I antibody for 45 min at 4°C. Subsequently cells were washed twice with PBS and incubated with a Cy2-conjugated secondary anti-mouse antibody for 45 min and analyzed using a FACSaria III (BD).

Electron microscopy
Piliation of gonococci was monitored by transmission electron microscopy. Gonococci were suspended in PBS, adsorbed to carbon-coated copper grids and fixed with 2% PFA for 15 min at RT. After washing several times with PBS and water the grids were incubated with 2% uranylacetate for 3 min and subsequent with 2% phosphotungstic acid for 3 min. The samples were viewed and photographed in a Zeiss EM10. 

Purification of caveolin-rich membrane fractions
Chang cells grown to confluence in 150-mm dishes were used to prepare caveolin-enriched membrane as described [5]. Briefly, Chang cells were either infected with N927 with an MOI of 50 for 1 h or not infected under low phosphate conditions. After washing with ice-cold phosphate-buffered saline, Chang cells (two confluent 150-mm dishes) were harvested into 2 ml of 500 mM sodium carbonate buffer, pH 11.0. Homogenization was carried out using a loose-fitting Dounce homogenizer (10 strokes) and a sonicator (three 20-s bursts). The homogenate was then suspended to 45% sucrose by the adding 2 ml of 90% sucrose prepared in MBS (25 mM Mes, pH 6.5, 0.15 M NaCl) and placed at the bottom of an ultracentrifuge tube. A 5-35% discontinuous sucrose gradient was formed above (4 ml of 5% sucrose/4ml of 35% sucrose; both in MBS containing 250 mM sodium carbonate) and centrifuged at 39,000 rpm for 24 h in a SW40 rotor. For the immunoblotting gradient fractions from the top of each gradient, 1-ml gradient fractions were collected to yield a total of 12 fractions. 

Cytotoxicity assay
Chang Cells cultured to 70% confluence were treated with the indicated concentrations of inhibitors or DMSO as a control for 1 h. Cells were collected and washed twice with phosphate-buffered saline (PBS) and resuspended in 100 ìl binding buffer (10 mM Hepes, 140 mM NaCl, 5 mM CaCl2, pH 7.4 containing 1 ìg/ml Propidium iodide (PI) for 10 min at 4 °C in the dark. As negative control 0.1% Triton was added 5 min before analysis. The cells were analyzed using a FACS Accuri C6.

References
1. Boettcher JP, Kirchner M, Churin Y, Kaushansky A, Pompaiah M, et al. (2010) Tyrosine-phosphorylated caveolin-1 blocks bacterial uptake by inducing Vav2-RhoA-mediated cytoskeletal rearrangements. PLoS Biol 8.
2. Kuhlewein C, Rechner C, Meyer TF, Rudel T (2006) Low-phosphate-dependent invasion resembles a general way for Neisseria gonorrhoeae to enter host cells. Infect Immun 74: 4266-4273.
3. Storz P, Toker A (2003) Protein kinase D mediates a stress-induced NF-kappaB activation and survival pathway. EMBO J 22: 109-120.
4. Wiznerowicz M, Trono D (2003) Conditional suppression of cellular genes: lentivirus vector-mediated drug-inducible RNA interference. J Virol 77: 8957-8961.
5. Song KS, Li S, Okamoto T, Quilliam LA, Sargiacomo M, et al. (1996) Co-purification and direct interaction of Ras with caveolin, an integral membrane protein of caveolae microdomains. Detergent-free purification of caveolae microdomains. J Biol Chem 271: 9690-9697.


Table S1: Primary antibodies for immunoblotting (IB), immunofluorescence (IF), immunoprecipitation (IP) or flow cytometry (FACS)
Antibody	Origin	Supplier	
Actin-â (AC-15)	monoclonal mouse IgG1	Sigma Aldrich A5441	
Active Rac1	monoclonal mouse 	New East Bioscience 26903	
AKT	monoclonal rabbit IgG	Cell Signaling 4685	
Caveolin	polyclonal rabbit	BD Transduction 610059	
Flotillin	polyclonal rabbit	Cell Signaling 3253	
GFP	mouse IgG2a	Santa Cruz sc-9996	
HA	monoclonal mouse IgG1	Cell Signaling 2367	
N. gonorrhoeae	polyclonal rabbit	US Biological N0600-02	
pAKT (Thr308)	monoclonal rabbit IgG	Cell Signaling 2965	
PI3K-p85	polyclonal rabbit 	Cell Signaling 4292	
PLCã1	monoclonal rabbit IgG	Cell Signaling 5690	
PI3K-p110	polyclonal rabbit IgG	Santa Cruz sc-7189	
PKD1/PKCµ	polyclonal rabbit 	Cell Signaling 2052	
pPKD1	polyclonal rabbit	Cell Signaling 2054	
Rac1	polyclonal rabbit	New East Bioscience 21003	
SREC-I 	polyclonal rabbit IgG	Santa Cruz sc-25483	
SREC-I	polyclonal goat IgG	Imagenex IMG-3370	
SREC-I 	monoclonal mouse IgG2b	R&D Systems MAB2409	
Tubulin (H-235)	polyclonal rabbit IgG	Santa Cruz 9104	
Vav2	monoclonal rabbit IgG	Cell Signaling 2848	
